# Optimal Exercise Dose for Improving Multidimensional Health Outcomes in Colorectal Cancer Patients: A Systematic Review, Meta‐Analysis and Meta‐Regression

**DOI:** 10.1002/ejsc.70283

**Published:** 2026-11-02

**Authors:** Jingyu Wang, Yuxuan He, Zhouluo Wang, So‐Eun Lee, Yoo‐Jin Shim, Si‐Eun Lee, Hye‐Ryeon Kim, Kyung‐Hee Lee, Ziqian Wang, Yongqi Miao, Seung‐Wook Choi

**Affiliations:** ^1^ School of Sports Science Qufu Normal University Qufu China; ^2^ Department of Sport Leisure Sungshin Women's University Seoul Republic of Korea; ^3^ School of Education and Arts Jiujiang Polytechnic University of Science and Technology Jiujiang China; ^4^ School of Philosophy and Sociology Jilin University Changchun China; ^5^ College of Wesley Creative Convergence Hyupsung University Hwaseong‐si Republic of Korea; ^6^ Department of Sports Science Chungwoon University Hongseong‐eup Republic of Korea; ^7^ Department of Exercise Therapy Gachon University Seoul Republic of Korea; ^8^ College of Physical Education Jilin University Changchun China

**Keywords:** colorectal neoplasms, drug therapy, exercise therapy, preoperative exercise

## Abstract

This study aimed to investigate the dose–response relationships between exercise prescription parameters and a range of health outcomes in patients with colorectal cancer (CRC). The following PRISMA guidelines, a systematic search of five databases was conducted. The inclusion criteria were randomized controlled trials (RCTs) of exercise interventions lasting ≥ 4 weeks in patients with CRC. Robust Variance Estimation (RVE) was used to synthesize the overall effects of exercise interventions across six outcome domains, including cardiorespiratory fitness (CRF) and muscular fitness (MF). Univariable RVE regression models and MetaForest analyses were employed to examine the moderating effects of two groups of variables: exercise prescription moderators and patient background characteristics. Risk of bias was assessed using the Cochrane tool, and the quality of evidence was evaluated using the GRADE approach. A total of 26 RCTs, involving 1731 CRC patients and 266 effect sizes, were included. CRF (ES = 0.35, *p* < 0.01) and MF (ES = 0.45, *p* < 0.01) showed statistically significant improvements. Regression analyses revealed threshold effects for both CRF and MF, with maximal improvements requiring sustained engagement in moderate‐to‐high intensity exercise. Subgroup analyses indicated that prehabilitation interventions were the most effective, while interventions during active treatment exhibited a decline–rebound trend over time. Based on these findings, high‐frequency and moderate‐to‐high volume interventions with individualized load regulation are recommended during the pre‐treatment phase. During active treatment, a more cautious approach is advised, involving reduced exercise volume during peak symptom periods and gradual progression during recovery.

## Highlights

- Exercise improved multidimensional health outcomes in CRC patients, with the strongest effects on cardiorespiratory fitness (CRF; ES = 0.35) and muscular fitness (MF; ES = 0.45) across 26 RCTs (*n* = 1731; 266 effect sizes).
- Across all stages, available evidence indicates that CRF/MF gains peak at a higher‐dose threshold (≥ 1400 MET‐min/week; ≤ 250 MET‐min/session; 5–7 sessions/week).
- Prehabilitation showed the strongest overall effects; during active treatment, the effects were weaker and time‐varying, supporting conservative, symptom‐adapted prescriptions.

**Abbreviations**
BC: body composition
BMI: body mass index
CAB: cancer‐associated biomarkers
CRC: colorectal cancer
CRF: cardiorespiratory fitness
HIIT: high‐intensity interval training
HRQoL: health‐related quality of life
MF: muscular fitness
MICT: moderate‐intensity continuous training
PFC: physical function capacity
RCTs: randomized controlled trials
RVE: robust variance estimation

## 1 Introduction

According to the 2022 report by the International Agency for Research on Cancer, colorectal cancer (CRC) ranks third in incidence and second in mortality among 36 types of cancer (Morgan et al. 2023). By 2050, the global burden of CRC is projected to reach approximately 3.1 million cases, with an estimated 1.68 million deaths (Ferlay et al. 2024). Furthermore, more than 30% of patients are at risk of disease recurrence (Howlader et al. 2014). Surgery, radiotherapy, and chemotherapy remain the most common treatments for CRC (Dekker et al. 2019). However, postoperative complications, chemotherapy‐related toxicity, and adverse side effects continue to pose substantial survival challenges for CRC patients, particularly within the first 3 years after diagnosis (Denlinger and Barsevick 2009). Therefore, effective strategies such as physical activity, pain management, and psychosocial support are essential to improve quality of life while maintaining optimal nutritional and physical status in CRC patients (Dekker et al. 2019).

Exercise has emerged as a promising strategy for survivorship care in CRC patients (Amirsasan et al. 2022; Q. Wang and Zhou 2021), with its safety well established in this population (Rock et al. 2022; Singh et al. 2020). Several systematic reviews and meta‐analyses have demonstrated the beneficial effects of exercise interventions on various health outcomes and clinical indicators across different treatment stages (Singh et al. 2020; Lund et al. 2020; Kraemer et al. 2022; Falz et al. 2022; Zhou et al. 2024; Machado et al. 2022; Liao et al. 2025; Gillis et al. 2018). Recent evidence suggests that structured exercise initiated within 6 months after adjuvant chemotherapy improves disease‐free survival in colon cancer patients (Courneya et al. 2025). However, in the context of CRC, the relationship between exercise prescription parameters and health outcomes remains insufficiently supported by systematic evidence (Campbell et al. 2019; Hayes et al. 2019). The use of generic exercise prescriptions may obscure the full therapeutic potential of exercise in oncology (Sasso et al. 2015). Precision in prescribing exercise parameters is essential to minimize harm and optimize health benefits (Sasso et al. 2015).

Early meta‐analyses have suggested a dose‐dependent relationship between exercise intensity and health outcomes (Cramer et al. 2014). Epidemiological studies have similarly reported a dose–response association between physical activity and CRC prevention (Wolin et al. 2009). However, determining the optimal exercise dosage for CRC patients remains challenging. This challenge stems from the inadequate reporting of exercise dosage in primary RCTs and the limitations of conventional statistical methodologies (Jetten et al. 2025).

Moreover, existing meta‐analyses have commonly employed subgroup analyses or conventional meta‐regression to examine the effects of exercise dosage on specific health outcomes in subsets of CRC patients. For example, interventions lasting longer than 12 weeks have been associated with greater improvements in quality of life, cardiorespiratory fitness, and reductions in body fat (Singh et al. 2020). Despite these findings, traditional meta‐regression approaches are limited by multicollinearity and the risk of overfitting, which hinder a more detailed exploration of heterogeneity sources (Parr et al. 2022). Subgroup analyses also depend on the dichotomization of continuous variables, which restricts interpretability. These methodological constraints significantly limit the ability to investigate exercise prescription parameters in depth and impede the development of precision‐based intervention strategies for CRC patients.

To overcome these methodological limitations, recent meta‐analyses have begun to adopt more flexible and robust modeling approaches. For instance, Robust Variance Estimation (RVE) offers a flexible meta‐regression framework with random effects that accommodates complex data structures (Tanner‐Smith et al. 2016). It enables the modeling of dependent effect sizes within the same study, even when the underlying correlation structure is unknown (Tipton 2015; Hedges et al. 2010). RVE has been applied to evaluate the effects of psychotherapy on psychological and functional outcomes among cancer survivors (Telles et al. 2024; Blumenstein et al. 2022). MetaForest, a machine learning technique that integrates random forest algorithms into meta‐analytic models, enables the identification of nonlinear relationships and complex interactions among moderator variables (Van Lissa 2020). To date, only one study in the exercise medicine field has applied a combined RVE and MetaForest approach to evaluate the effects of regular exercise on metabolic and inflammatory biomarkers in cancer patients (J. Wang, He, et al. 2025).

Therefore, this study employed a dual‐modeling strategy combining RVE and MetaForest to systematically synthesize existing RCT evidence and examine dose–response trends of exercise prescription parameters across multiple dimensions of health outcomes. The aim is to provide an evidence‐based foundation for individualized exercise prescription in CRC patients. Specifically, the objectives were: (1) to synthesize effect sizes using RVE and explore linear associations between moderator variables and intervention effects; (2) to assess the importance and nonlinear trends of moderators using MetaForest; and (3) to perform subgroup analyses based on cancer treatment stage.

## 2 Methods

This systematic review has been registered in PROSPERO (Registration number: CRD42024607473) and was conducted according to the PRISMA guidelines (Supporting Information S1) (Page et al. 2021).

### 2.1 Data Sources, Literature Search, and Inclusion Criteria

A systematic search was conducted from database inception until November 6, 2024, across five databases: PubMed (MEDLINE), Embase, Cochrane Central Register of Controlled Trials (CENTRAL), SPORTDiscus, and Web of Science. Additionally, the reference lists of included articles and previous systematic reviews or meta‐analyses were checked to identify relevant studies. The search strategy was based on the PICOS principles (Supporting Information S1).

The specific inclusion criteria were as follows: population: CRC patients aged 18 years and older; intervention: exercise interventions (including those explicitly reporting exercise parameters to enhance physical activity levels); comparison: non‐exercise control groups (including standard care, health education, or wait‐list controls); outcomes: at least one health‐related indicator measured as an outcome; study design: randomized controlled trials (RCTs).

The exclusion criteria were as follows: population: non‐CRC patients; intervention: rehabilitation exercises (e.g., pelvic floor muscle training and hand‐foot exercises) and mind‐body exercises (e.g., yoga, tai chi), as well as interventions lasting less than 4 weeks; control: studies without a control group; outcomes: surgical‐related indicators (e.g., perioperative indicators, tumor resection quality, postoperative recovery, and long‐term prognosis); study design: systematic reviews, narrative reviews, animal studies, and non‐English studies. Studies were also excluded if full text or relevant data were unavailable even after contacting the authors.

### 2.2 Study Selection and Data Extraction

Two authors (Wang and He) independently reviewed studies to identify potentially eligible trials that met the inclusion criteria. In cases of disagreement, a third experienced author (Kim) resolved the issue. The following study characteristics were extracted: basic study information (first author, study registration number), outcome measurements (excluding follow‐up data), and moderator variables. For studies reporting data in non‐mean ± SD formats such as median (quartiles), SE, or *p*‐values for pre‐ and post‐intervention, data were converted according to the Cochrane Handbook for Systematic Reviews of Interventions (Cumpston et al. 2019).

#### 2.2.1 Classification of Health Outcome Indicators

Referring to the American College of Sports Medicine Guidelines for Exercise Testing and Prescription and outcomes reported in RCTs of exercise interventions in CRC patients, health outcome indicators were categorized into six domains: body composition (BC), cancer‐associated biomarkers (CAB), cardiorespiratory fitness (CRF), muscular fitness (MF), physical function capacity (PFC), and health‐related quality of life (HRQoL) (Liguori 2020). Detailed classification information is provided in Supporting Information S1.

#### 2.2.2 Classification and Processing of Moderator Variables

In this study, 25 potential moderator variables relevant to exercise interventions were prespecified. Moderator variables with a missing rate greater than 30% were excluded, while those with a missing rate between 0% and 30% were imputed using multiple imputation. Based on their functional roles, moderators were categorized into two groups: (1) exercise prescription moderator variables (e.g., type, duration, frequency, intensity per session, total volume), which served as the primary focus of the analysis; and (2) background moderator variables (e.g., age, male ratio, BMI), which were used to account for demographic and study design heterogeneity. This classification scheme was intended to clarify the study's analytical focus while controlling for potential confounding factors that could obscure the interpretation of intervention effects.

### 2.3 Risk of Bias and Quality of Evidence Assessment

The risk of bias in the included RCTs was assessed using the Cochrane Risk of Bias tool (RoB 2). Two authors (Wang and He) independently evaluated the risk of bias, and disagreements were resolved by a third reviewer (Kim). Inter‐rater agreement was acceptable (Kappa = 0.817). The quality of evidence was assessed using the GRADE (Grading of Recommendations, Assessment, Development and Evaluation) framework.

### 2.4 Data Analysis

All statistical analyses were performed using R (version 4.4.1) (Team 2024). Cohen's d and its variance were used as effect size metrics suitable for small‐sample studies. A d of 0.2 indicates a small effect, 0.5 a moderate effect, and 0.8 a large effect (Cohen 2013). All effect sizes were transformed so that positive values indicated beneficial outcomes. Moderator variables with missing data were imputed using the “mice” package (Van Buuren and Groothuis‐Oudshoorn 2011). Meta‐regression models were then fitted using the “metafor” package, and variance inflation factors (VIF) were calculated to assess multicollinearity. Moderators with VIF > 10 were excluded (Akinwande et al. 2015).

The RVE method was applied using the “robumeta” package (Fisher and Zhipeng 2023). The regression model adopted a correlated effects structure, assuming an intra‐study correlation of 0.5 (model weight = “CORR”, rho = 0.5). Sensitivity analyses were performed by varying rho from 0.1 to 0.9 in increments of 0.05 (e.g., rho = 0.1, 0.15, 0.2). The overall mean effect size was estimated using an RVE meta‐regression model without covariates. Between‐study heterogeneity was assessed using the I^2^ statistic, with I^2^ > 25% indicating low, > 50% moderate, and > 75% high heterogeneity. Egger's test was performed using the “metafor” package to detect publication bias, and the Trim‐and‐Fill method was used to adjust for potential bias and generate corrected funnel plots (Duval and Tweedie 2000; Egger et al. 1997). Univariate RVE regression models were then fitted, and each model's I^2^ and *R* ^2^ were recorded. In RVE models, *R* ^2^ represents the proportion of heterogeneity explained by the moderators. The model with *R* ^2^ closest to 1 was considered optimal (Plonsky and Ghanbar 2018).

MetaForest was conducted following the method proposed by Van Lissa (Van Lissa 2020). Random‐effect weights (“whichweights” = “random”) were applied, with 20,000 decision trees. Convergence plots were visualized. Cross‐validation and parameter tuning were conducted using the “caret” package with 100 bootstrap resampling iterations. Bootstrap ensures robustness, particularly in small‐sample contexts. Parameters tuned included weight type (random, fixed, unif), number of splitting variables (mtry = 2, 4, 6), and minimum node size (min.node.size = 2, 4). Performance metrics such as *R* ^2^_CV_ (cross‐validated *R* ^2^) were extracted for the optimal model. Partial dependence plots and variable importance plots were generated. In MetaForest, *R* ^2^ primarily reflects model prediction accuracy and generalizability. Since the aim was to explore the contribution of moderators to effect sizes, emphasis was placed on *R* ^2^_CV_. Subgroup MetaForest analyses were conducted based on intervention timing (during chemotherapy, prehabilitation, and survivorship), with model parameters unchanged. Data visualization was conducted using the “ggplot2” package (Wickham 2016).

## 3 Results

A total of 9408 abstracts were initially identified, of which 70 were deemed potentially relevant. After full‐text screening, 44 studies were excluded, and 7 additional studies were identified through other sources. Ultimately, 33 studies were included, reporting data from 26 RCTs (Supporting Information S1). One RCT was a multi‐arm trial from which two separate effect sizes were extracted, each comparing an intervention group to the control group. The complete screening and selection process is illustrated in Figure 1.

**FIGURE 1:**
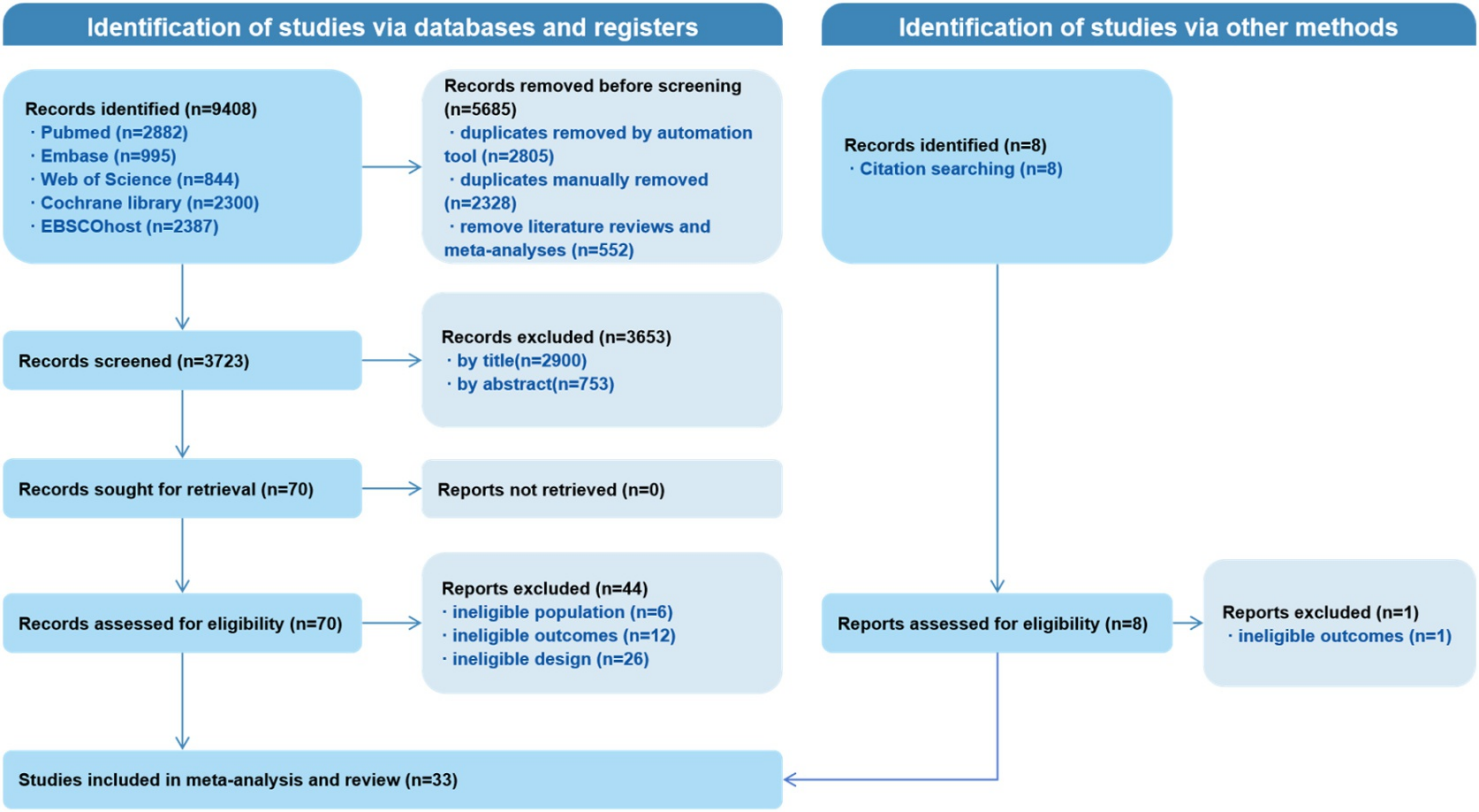
Preferred reporting items for systematic reviews and meta‐analyses (PRISMA) flow diagram of record identification, screening, and selection processes.

### 3.1 Overall Effect, Risk of Bias, and Publication Bias

The 26 RCTs included a total of 1731 CRC patients, with 829 in the control groups and 902 in the intervention groups. Exercise‐related adverse events were primarily limited to joint and muscle pain, and no RCT reported any serious exercise‐related adverse events. Figure 2 presents the synthesis of 266 effect sizes derived from the 26 RCTs. The overall effect size was ES = 0.20 (SE = 0.03, *p* < 0.01, I^2^ = 30.1%). Among the health outcomes, CRF (ES = 0.35, SE = 0.06, *p* < 0.01, I^2^ = 5.74%) and MF (ES = 0.45, SE = 0.10, *p* < 0.01, I^2^ = 47.2%) demonstrated significant and moderate effects (Supporting Information S1: Table S1). Sensitivity analyses indicated that setting rho = 0.5 did not affect model stability (Supporting Information S1: Table S2).

**FIGURE 2:**
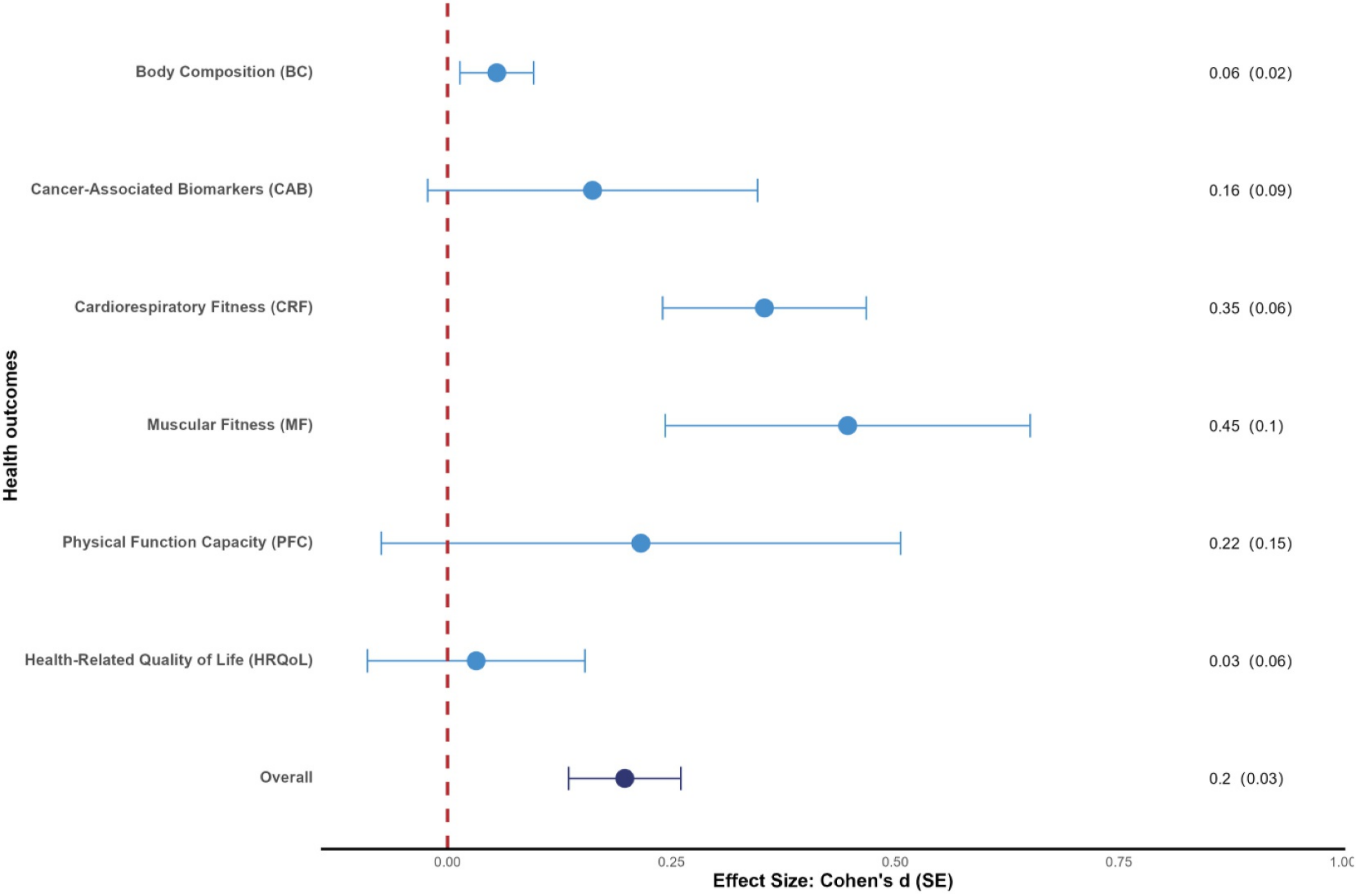
Forest plot for meta‐analyses.

Risk of bias was assessed at the outcome level using the Cochrane RoB 2 tool. Study‐level summaries are presented in (Supporting Information S1: Figure S1 and S2), with supporting justifications in Supporting Information S1: Table S3 and assessments across the six outcome domains in Supporting Information S1: Figure S3. Based on the highest risk‐of‐bias judgment among the assessed outcomes within each study, 19 trials (73.1%) were classified as having some concerns and 7 trials (26.9%) as having high risk of bias. Concerns were most frequent in outcome measurement and selection of the reported result, followed by missing outcome data and the randomization process.

Funnel plots for the overall analysis and the six outcome domains are presented in (Supporting Information S1: Figure S4 and S5). Egger's test yielded *p* = 0.0566 for the overall analysis and *p* = 0.0861 for physical function capacity, whereas *p*‐values exceeded 0.10 for the other five outcome domains.

### 3.2 Results of Moderator Analysis

Among the 25 prespecified potential moderator variables, 15 met the criteria for multiple imputation and multicollinearity testing and were included in the final analysis (Supporting Information S1: Tables S4 and S5). The linear moderating effects of the 15 variables were explored for six outcome categories using RVE univariable regression analysis. RVE models that produced significant moderator effects and reduced heterogeneity are reported in Supporting Information S1. Supporting Information S1 provides the parameter estimates of 12 MetaForest models (Supporting Information S1: Table S6) and model convergence diagnostics (Supporting Information S1: Figure S6 and S7), all of which indicated satisfactory convergence.

Figure 3 presents the variable importance rankings of exercise prescription moderators as identified by the MetaForest model. Supporting Information S1: Figure S8–S13 display partial dependence plots illustrating the nonlinear trends of each moderator. For CRF and MF, the optimal exercise dose range was identified as ≥ 1400 MET‐min/week, ≤ 250 MET‐min/session, and 5–7 sessions/week, with supervised interventions outperforming unsupervised ones. For PFC, combined training (aerobic plus resistance training) yielded the greatest effects. For BC and CAB, the MetaForest model did not identify any exercise prescription parameters as important moderators. No clear nonlinear patterns were observed for HRQoL.

**FIGURE 3:**
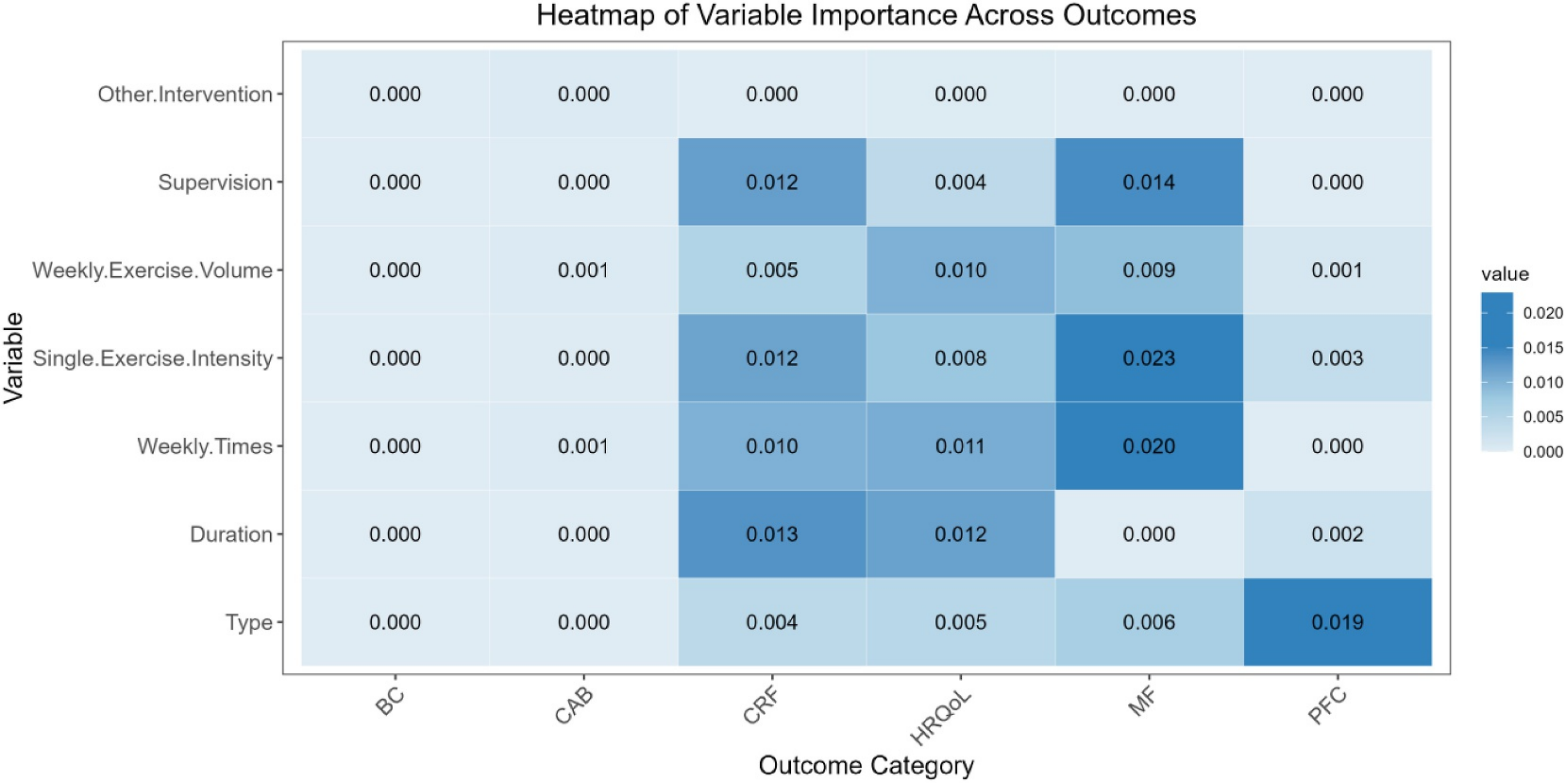
Heatmap of variable importance for exercise prescription moderators across the six outcome domains, generated using MetaForest.

Supporting Information S1: Figure S14 shows the variable importance rankings of background moderators generated by the MetaForest model, and Supporting Information S1: Figure S15–S20 present their partial dependence plots. Among these, body weight and the colon cancer ratio showed notable moderating effects on MF. Other background variables, such as intervention timing, patient weight, and age, also exhibited moderating effects on specific outcome domains.

### 3.3 Subgroup Analysis

Subgroup analyses were conducted based on the timing of exercise intervention, and the results are presented in Supporting Information S1. Overall, prehabilitation interventions produced the most favorable effects (ES = 0.24, SE = 0.07, *p* < 0.01, I^2^ = 30.54%), followed by interventions in CRC survivors (ES = 0.18, SE = 0.03, *p* < 0.01, I^2^ = 38.89%) and those undergoing chemotherapy (ES = 0.15, SE = 0.11, *p* = 0.25, I^2^ = 22.91%) (Supporting Information S1: Figure S21, Table S7). Due to the reduced number of effect sizes within each subgroup, additional MetaForest analyses were performed across the full sample. Detailed MetaForest model parameters are presented in Supporting Information S1: Table S8.

Partial dependence plots revealed the nonlinear trends of each moderator within cancer treatment subgroups (Supporting Information S1: Figure S22–S27). In the Chemotherapy subgroup, duration and weekly exercise volume emerged as significant exercise prescription moderator variables (Supporting Information S1: Table S9). The effect size showed a declining trend between 8 and 20 weeks, followed by a gradual increase from 20 to 24 weeks. No additional benefit was observed when the total exercise volume exceeded 500 MET‐min/week or when the intensity per session exceeded 180 MET‐min. A frequency of 3 times/week was sufficient to elicit maximal improvements. In contrast, the Preoperative subgroup demonstrated that a total volume greater than 1300 MET‐min/week and a frequency of 6–7 times/week were necessary to achieve optimal effects.

### 3.4 Quality of Evidence Assessment

The quality of evidence was evaluated using the GRADE framework, and the results are summarized in Supporting Information S1. The synthesized effect sizes were rated from “Moderate” to “Very Low” (Supporting Information S1: Table S10). The MetaForest models were rated from “Low” to “Very Low” (Supporting Information S1: Table S11).

## 4 Discussion

Building on evidence supporting the benefits of exercise interventions for multidimensional health outcomes in CRC patients, this meta‐analysis further explored the dose–response relationships between exercise prescription parameters and those outcomes. The overall findings suggest that CRC patients may derive multidimensional health benefits from exercise across different treatment phases, with the most pronounced effects observed in cardiorespiratory fitness (CRF) and muscular fitness (MF). Regression analyses revealed threshold effects, indicating that maximal improvements in CRF and MF depend on sustained engagement in moderate‐to‐high intensity exercise. Subgroup analyses further demonstrated that prehabilitation interventions yielded the most favorable effects, while those delivered during active treatment exhibited a decline–rebound trend over time. Based on these findings, we recommend tailoring exercise prescriptions to the patient's specific treatment or recovery stage. These results highlight the complexity of dose–response relationships across treatment phases and outcome domains in CRC care and provide meaningful insights for designing personalized exercise strategies in clinical settings.

Our findings primarily identify the optimal dose range for improving CRF and MF in CRC patients. Previous meta‐analyses in cancer populations have reported that higher exercise doses are significantly associated with greater improvements in CRF and MF (Bjørke et al. 2019; Sweegers et al. 2019). Our study supports and refines these findings, suggesting an effective threshold of approximately 1400 MET‐min/week for both outcomes. Based on our calculations, current exercise guidelines for cancer patients—which recommend at least 150 minutes of moderate‐intensity aerobic exercise and two sessions of resistance training per week—correspond to a minimum of about 800 MET‐min/week (Campbell et al. 2019; Hayes et al. 2019). This suggests that the lower boundary of current guidelines may fall short of the optimal dose needed to elicit maximal gains in CRF and MF.

With regard to exercise intensity, some studies have highlighted the potential advantages of high‐intensity exercise. For example, one RCT showed that a 4‐week high‐intensity program (85%–95% HRmax) led to greater improvements in CRF compared to a moderate‐intensity program (70% HRmax) (Devin et al. 2016). Similarly, a meta‐analysis concluded that at least 20 minutes of high‐intensity training is required to produce meaningful improvements in CRF among cancer patients (Lavín‐Pérez et al. 2021). However, our findings suggest that the benefits of increasing intensity reach a plateau beyond a certain threshold. For instance, a study of CRC survivors reported high overall adherence to exercise prescriptions, yet only 28.9% adhered to the high‐intensity interval training (HIIT) component, compared to 95.7% adherence to moderate‐intensity continuous training (MICT) (Moraitis et al. 2023). Similar patterns were observed in breast cancer patients, where adherence to HIIT was lower than to MICT (Witlox et al. 2019). These findings highlight the potential trade‐off between physiological gains and adherence. Moreover, high‐intensity exercise may be more beneficial for maintaining long‐term outcomes rather than maximizing short‐term effects (Devin et al. 2018; Minnella et al. 2020). Therefore, we caution against indiscriminately increasing exercise intensity in clinical prescriptions. This recommendation is supported by RCT evidence suggesting that HIIT does not confer significantly greater improvements in MF compared with MICT among cancer survivors (Kampshoff et al. 2015).

Similarly, previous meta‐analyses that did not differentiate between cancer types reported no significant effects of exercise frequency on CRF during cancer treatment or significant effects of exercise prescription parameters (frequency, intensity, type, and duration) on health‐related quality of life (HRQoL) or physical function capacity (PFC) during or after treatment (Bjørke et al. 2019; Sweegers et al. 2018). However, the inclusion of heterogeneous cancer populations in those analyses may have obscured CRC‐specific responses. In contrast, our findings suggest that a training frequency of at least five sessions per week may be necessary to achieve improvements in CRF and MF among CRC patients. Notably, prior evidence has indicated a compensatory relationship between training frequency and session duration. For example, an 8‐week high‐frequency training program (two 35‐minute moderate‐intensity sessions and two 28‐minute high‐intensity sessions per week) produced comparable improvements in VO_2_max and other physiological indicators to a low‐frequency program (one 70‐minute moderate‐intensity session and one 56‐minute high‐intensity session per week) (Tripp et al. 2025). Therefore, while prescriptions should be stage‐tailored, the overall pattern suggests that higher‐frequency, moderate‐to‐high intensity exercise is most consistently associated with greater CRF and MF gains in CRC patients. According to our calculation method, this may be achieved through approximately 40‐minute sessions of moderate‐to‐high intensity aerobic exercise (heart rate reserve, HRR > 70%) or 25‐minute sessions of high‐intensity aerobic exercise (HRR > 85%), performed five times per week, combined with two to three sessions of resistance training.

In addition, we found that both semi‐supervised and fully supervised interventions were associated with greater benefits. This is consistent with previous meta‐analyses showing that supervised exercise is more effective in sustaining long‐term physical activity, enhancing HRQoL, and reducing fatigue among CRC survivors (Agirre‐Elordui et al. 2024). Qualitative research has further shown that patients with advanced cancer often prefer professional supervision by exercise specialists with oncology training, to ensure safe implementation, proper monitoring, and progressive adjustment of exercise programs (Bland et al. 2022). Interestingly, our findings indicated that semi‐supervised interventions were more effective than fully supervised programs in improving CRF. This suggests that in resource‐constrained clinical settings, semi‐supervised approaches may offer a more feasible and scalable method for exercise delivery. This aligns with recent recommendations from integrated care models, which advocate embedding exercise services into cancer care through stage‐based referral pathways and individualized programming tailored to patients' functional status and treatment phase (Cheema et al. 2019).

Building on these findings, we further examined how to optimize exercise prescriptions across different phases of cancer treatment in CRC patients. Our analysis indicated that exercise interventions implemented during the prehabilitation phase produced the most favorable overall effects compared with other treatment stages. Existing evidence highlights the benefits of prehabilitation, showing that incorporating exercise into preoperative programs can enhance PFC and reduce postoperative hospital stays in CRC patients (Molenaar et al. 2022; Marmol‐Perez et al. 2024). Previous studies have shown that both HIIT and MICT can improve preoperative functional capacity in CRC patients, although HIIT may lead to superior long‐term physical health benefits 2 months post‐surgery (Minnella et al. 2020). Recent prehabilitation guidelines for cancer further indicate that exercise interventions as short as 2 weeks may be beneficial, with greater effects observed when interventions exceed 4 weeks, and emphasize the implementation of individualized, moderate‐to‐high intensity programmes centered on combined aerobic and resistance exercise within the limited preoperative window (Support 2025). Consistent with these recommendations, our results further suggest that during the prehabilitation phase, intervention effects were optimized when total weekly exercise volume exceeded 1300 MET‐min and training frequency reached 6 to 7 sessions per week. Based on these insights, we recommend that prehabilitation programs prioritize high‐frequency, moderate‐to‐high volume interventions incorporating individualized load regulation. Such strategies aim to enhance CRF and MF within the limited preoperative window and lay a strong foundation for postoperative recovery and long‐term survivorship.

In contrast, exercise interventions implemented during the active treatment phase produced the weakest overall effects among all treatment stages. The MetaForest model further identified intervention duration and total weekly volume as key moderators influencing effectiveness during this phase. A declining trend in effect size was observed between weeks 8 and 20, followed by a gradual rebound between weeks 20 and 24. This pattern may be attributed to treatment‐related adverse effects that impair patients' physiological capacity to adapt to exercise during early intervention periods, thereby diminishing its efficacy (Eng 2009; Hamaker et al. 2015). As treatment progresses, some patients may complete primary therapeutic regimens or adapt to treatment side effects, which may allow for partial recovery of exercise‐related benefits.

Notably, increasing training loads—such as session intensity exceeding 180 MET‐min, weekly volume over 500 MET‐min/week, or frequency above three sessions per week—did not produce additional benefits during this stage. Previous studies have suggested that conventional, standard linear progressive training models, though safe and easy to implement, may be insufficient to accommodate the dynamic physiological fluctuations induced by cancer treatments (Sasso et al. 2015). In response, a chemotherapy‐periodized exercise model has been proposed, which involves proactively reducing training volume and intensity during symptom peaks and gradually restoring load during recovery phases (Kirkham et al. 2020). Trials based on this approach have shown that adherence is lowest when exercise is performed three to 5 days post‐chemotherapy, while adherence improves when training resumes during recovery (Kirkham et al. 2020). More recent randomized evidence further indicates that higher exercise adherence is associated with reductions in circulating tumor cells in patients with colorectal cancer (Brown et al. 2025). Based on these findings, we recommend adopting a conservative approach to exercise prescription during active treatment in CRC patients. Training volume, intensity, and frequency should be actively reduced during periods of peak treatment‐related toxicity, and program complexity minimized. Gradual progression in training load should then be introduced during recovery. GRADE evaluation of the exercise interventions included in this study indicated that the quality of evidence for most primary outcomes ranged from moderate to very low, suggesting that a degree of uncertainty remains in the current conclusions. This consideration is particularly critical during the active treatment phase, where careful adjustment of exercise parameters is essential to ensure patient safety. For high‐risk patients—such as those with severe comorbidities—exercise prescriptions should be tailored based on professional assessment (Campbell et al. 2019).

Beyond CRF and MF, no stable or consistent dose–response relationships were identified for other outcomes in the present study. This may be attributable to heterogeneity in the included interventions, outcome‐specific physiological sensitivity, and variability in baseline patient characteristics. Prior evidence indicates that exercise intensity is a key driver of changes in cancer‐associated biomarkers (IL‐6, TNF‐α, CRP) (J. Wang, He, Wang, et al. 2025; J. Wang, He, Kim, et al. 2025), but the limited number of high‐intensity interventions in this analysis may have hindered the detection of dose–response effects. In addition, randomized trials in CRC survivors have shown that different aerobic exercise doses (e.g., 150 vs. 300 minutes per week) do not result in significantly different biomarker responses, indicating that increasing exercise duration alone may not elicit linear biological effects (Brown, Troxel, et al. 2018; Brown, Rickels, et al. 2018; Brown, Rhim, et al. 2018, 2023). Consistent with this finding, exercise intensity does not appear to yield clinically meaningful differences in cancer‐related fatigue (Kampshoff et al. 2015; Demmelmaier et al. 2021). Moreover, during the perioperative and chemotherapy phases, patients with poorer baseline functional status tend to experience greater improvements in physical function following exercise interventions (Brown et al. 2024; Minnella et al. 2016). Finally, in advanced CRC, higher baseline inflammation may increase the apparent responsiveness of biomarkers to exercise (Brown et al. 2023).

Several limitations of this study warrant attention. First, the number of available RCTs was insufficient to support a more granular classification of effect sizes. For example, cancer‐associated biomarkers could have been further stratified into inflammatory and metabolic biomarkers, while HRQoL could have been decomposed into distinct prognostic domains. Second, we did not observe clear dose–response relationships for outcomes other than CRF and MF, which limits the ability to recommend optimal ranges for additional health domains. Third, our analyses were based on aggregate rather than individual patient data (IPD), which restricted our ability to adjust for confounding at the patient level. Fourth, although recent evidence suggests that short‐term exercise interventions (e.g., 2–4 weeks) during the prehabilitation period may confer certain benefits, the current evidence base was insufficient to support an evaluation of dose–response effects for such short‐duration interventions. Fifth, to comprehensively assess the effects of exercise across multiple health domains and to explore dose–response patterns at the construct level, we pooled different tests intended to capture the same broad outcomes and summarized the effects using Cohen's d. Although this approach was necessary in the absence of standardized outcome measures, heterogeneity in test sensitivity, learning effects, and measurement error may have introduced uncertainty into pooled estimates. Sixth, although Egger's tests were largely non‐significant, results should be interpreted cautiously, as funnel plot asymmetry may reflect heterogeneity in outcome measurement and test responsiveness rather than publication bias per se. Furthermore, the GRADE evaluation indicated moderate to very low overall certainty of evidence and potential risk of bias, suggesting that the present findings should be interpreted with caution.

To address these limitations, several key directions for future research are proposed. First, additional high‐quality RCTs are needed across all phases of CRC care, particularly those incorporating frequent monitoring of intervention effects during active cancer treatment. Second, future trials should report exercise parameters and outcome measures in a standardized and transparent manner to enhance comparability across studies. Third, IPD meta‐analyses are needed to more precisely examine the moderating effects of patient characteristics on exercise outcomes. Fourth, future research should place greater emphasis on the potential health benefits of short‐term exercise interventions (< 4 weeks) during the prehabilitation period. Lastly, further research is warranted to clarify the biological mechanisms that underlie the effects of exercise interventions in CRC patients.

## 5 Conclusion

This study systematically examined the beneficial effects of exercise interventions on multidimensional health outcomes in patients with CRC and explored strategies to optimize exercise prescription parameters for maximal intervention efficacy. Overall, improvements in cardiorespiratory fitness and muscular health require sustained engagement in moderate‐to‐high intensity exercise. During the pre‐treatment phase, high‐frequency and moderate‐to‐high volume interventions with individualized load regulation are recommended. During active treatment, a more cautious approach may be appropriate, involving reductions in frequency, intensity, and volume during peak symptom periods, followed by gradual increases during recovery. To enhance the clinical feasibility and translational value of exercise interventions, future research should further elucidate the biological mechanisms by which exercise prescription parameters influence health outcomes in CRC patients.

## Author Contributions

All authors contributed to the study conception and design. Material preparation and data collection were performed by Jingyu Wang, Yuxuan He and Hye‐Ryeon Kim. Data analysis was conducted by Jingyu Wang, Ziqian Wang and Yongqi Miao. The first draft of the manuscript was written by Jingyu Wang and all authors commented on previous versions of the manuscript. All authors have read and approved the final manuscript.

## Funding

The authors have nothing to report.

## Ethics Statement

The authors have nothing to report.

## Consent

The authors have nothing to report.

## Conflicts of Interest

The authors declare no conflicts of interest.

## Preprint Statement

The authors confirm that this manuscript has not been posted on any preprint server.

## Supporting information


Supporting Information S1

## Data Availability

The data that support the findings of this study are available from the corresponding author upon reasonable request.
