# Supplementary material for: Optimal Exercise Dose for Improving Multidimensional Health Outcomes in Colorectal Cancer Patients: A Systematic Review, Meta‐Analysis and Meta‐Regression
Source: Eur J Sport Sci. 2026 Nov 2;26(11):e70283. doi: 10.1002/ejsc.70283 (PMC13725542; doi:10.1002/ejsc.70283)
Supplement: Supplementary file 1 [file EJSC-26-e70283-s001.docx]

**Supplementary information 1. PRISMA 2020 checklist.**

| **Section and Topic** | **Item #** | **Checklist item** | **Location where item is reported** |
| --- | --- | --- | --- |
| **TITLE** | | |  |
| Title | 1 | Identify the report as a systematic review. | 1 |
| **ABSTRACT** | | |  |
| Abstract | 2 | See the PRISMA 2020 for Abstracts checklist. | 1 |
| **INTRODUCTION** | | |  |
| Rationale | 3 | Describe the rationale for the review in the context of existing knowledge. | 3-5 |
| Objectives | 4 | Provide an explicit statement of the objective(s) or question(s) the review addresses. | 5 |
| **METHODS** | | |  |
| Eligibility criteria | 5 | Specify the inclusion and exclusion criteria for the review and how studies were grouped for the syntheses. | pp. 6–7; Supplementary Information 3 |
| Information sources | 6 | Specify all databases, registers, websites, organisations, reference lists and other sources searched or consulted to identify studies. Specify the date when each source was last searched or consulted. | 6 |
| Search strategy | 7 | Present the full search strategies for all databases, registers and websites, including any filters and limits used. | 6; Supplementary Information 2 |
| Selection process | 8 | Specify the methods used to decide whether a study met the inclusion criteria of the review, including how many reviewers screened each record and each report retrieved, whether they worked independently, and if applicable, details of automation tools used in the process. | 6-7 |
| Data collection process | 9 | Specify the methods used to collect data from reports, including how many reviewers collected data from each report, whether they worked independently, any processes for obtaining or confirming data from study investigators, and if applicable, details of automation tools used in the process. | 6-7 |
| Data items | 10a | List and define all outcomes for which data were sought. Specify whether all results that were compatible with each outcome domain in each study were sought (e.g. for all measures, time points, analyses), and if not, the methods used to decide which results to collect. | 7 |
|  | 10b | List and define all other variables for which data were sought (e.g. participant and intervention characteristics, funding sources). Describe any assumptions made about any missing or unclear information. | 7-8 |
| Study risk of bias assessment | 11 | Specify the methods used to assess risk of bias in the included studies, including details of the tool(s) used, how many reviewers assessed each study and whether they worked independently, and if applicable, details of automation tools used in the process. | 8 |
| Effect measures | 12 | Specify for each outcome the effect measure(s) (e.g. risk ratio, mean difference) used in the synthesis or presentation of results. | 8 |
| Synthesis methods | 13a | Describe the processes used to decide which studies were eligible for each synthesis (e.g. tabulating the study intervention characteristics and comparing against the planned groups for each synthesis (item #5)). | 6-7 |
|  | 13b | Describe any methods required to prepare the data for presentation or synthesis, such as handling of missing summary statistics, or data conversions. | 7-8 |
|  | 13c | Describe any methods used to tabulate or visually display results of individual studies and syntheses. | 9 |
|  | 13d | Describe any methods used to synthesize results and provide a rationale for the choice(s). If meta-analysis was performed, describe the model(s), method(s) to identify the presence and extent of statistical heterogeneity, and software package(s) used. | 8-9 |
|  | 13e | Describe any methods used to explore possible causes of heterogeneity among study results (e.g. subgroup analysis, meta-regression). | 8-9 |
|  | 13f | Describe any sensitivity analyses conducted to assess robustness of the synthesized results. | 8 |
| Reporting bias assessment | 14 | Describe any methods used to assess risk of bias due to missing results in a synthesis (arising from reporting biases). | 8 |
| Certainty assessment | 15 | Describe any methods used to assess certainty (or confidence) in the body of evidence for an outcome. | 8 |
| **RESULTS** | | |  |
| Study selection | 16a | Describe the results of the search and selection process, from the number of records identified in the search to the number of studies included in the review, ideally using a flow diagram. | 10 |
|  | 16b | Cite studies that might appear to meet the inclusion criteria, but which were excluded, and explain why they were excluded. | 10 |
| Study characteristics | 17 | Cite each included study and present its characteristics. | 10 |
| Risk of bias in studies | 18 | Present assessments of risk of bias for each included study. | 12 |
| Results of individual studies | 19 | For all outcomes, present, for each study: (a) summary statistics for each group (where appropriate) and (b) an effect estimate and its precision (e.g. confidence/credible interval), ideally using structured tables or plots. | 10 |
| Results of syntheses | 20a | For each synthesis, briefly summarise the characteristics and risk of bias among contributing studies. | 10 |
|  | 20b | Present results of all statistical syntheses conducted. If meta-analysis was done, present for each the summary estimate and its precision (e.g. confidence/credible interval) and measures of statistical heterogeneity. If comparing groups, describe the direction of the effect. | 10-12 |
|  | 20c | Present results of all investigations of possible causes of heterogeneity among study results. | 11-12 |
|  | 20d | Present results of all sensitivity analyses conducted to assess the robustness of the synthesized results. | 10 |
| Reporting biases | 21 | Present assessments of risk of bias due to missing results (arising from reporting biases) for each synthesis assessed. | 11 |
| Certainty of evidence | 22 | Present assessments of certainty (or confidence) in the body of evidence for each outcome assessed. | 12-13 |
| **DISCUSSION** | | |  |
| Discussion | 23a | Provide a general interpretation of the results in the context of other evidence. | 14-19 |
|  | 23b | Discuss any limitations of the evidence included in the review. | 18-20 |
|  | 23c | Discuss any limitations of the review processes used. | 19-20 |
|  | 23d | Discuss implications of the results for practice, policy, and future research. | 14-18, 20-21 |
| **OTHER INFORMATION** | | |  |
| Registration and protocol | 24a | Provide registration information for the review, including register name and registration number, or state that the review was not registered. | 6 |
|  | 24b | Indicate where the review protocol can be accessed, or state that a protocol was not prepared. | 6 |
|  | 24c | Describe and explain any amendments to information provided at registration or in the protocol. | 6 |
| Support | 25 | Describe sources of financial or non-financial support for the review, and the role of the funders or sponsors in the review. | Title Page |
| Competing interests | 26 | Declare any competing interests of review authors. | Title Page |
| Availability of data, code and other materials | 27 | Report which of the following are publicly available and where they can be found: template data collection forms; data extracted from included studies; data used for all analyses; analytic code; any other materials used in the review. | Title Page |

*From:* Page MJ, McKenzie JE, Bossuyt PM, Boutron I, Hoffmann TC, Mulrow CD, et al. The PRISMA 2020 statement: an updated guideline for reporting systematic reviews. BMJ 2021;372:n71. doi: 10.1136/bmj.n71. This work is licensed under CC BY 4.0. To view a copy of this license, visit

**Supplementary information 2. Search strategies.**

| Database | Search strategy |
| --- | --- |
| PubMed | #1. ((((((((((((((((((((((((((((((((((((((((((((((((((((((((Exercises[MeSH Terms]) OR (Exercise, Physical[Title/Abstract])) OR (Exercises, Physical[Title/Abstract])) OR (Physical Exercise[Title/Abstract])) OR (Physical Exercises[Title/Abstract])) OR (Physical Activity[Title/Abstract])) OR (Activities, Physical[Title/Abstract])) OR (Activity, Physical[Title/Abstract])) OR (Physical Activities[Title/Abstract])) OR (Exercise, Aerobic[Title/Abstract])) OR (Aerobic Exercise[Title/Abstract])) OR (Aerobic Exercises[Title/Abstract])) OR (Exercises, Aerobic[Title/Abstract])) OR (Exercise, Isometric[Title/Abstract])) OR (Exercises, Isometric[Title/Abstract])) OR (Isometric Exercises[Title/Abstract])) OR (Isometric Exercise[Title/Abstract])) OR (Acute Exercise[Title/Abstract])) OR (Acute Exercises[Title/Abstract])) OR (Exercise, Acute[Title/Abstract])) OR (Exercises, Acute[Title/Abstract])) OR (Exercise Training[Title/Abstract])) OR (Exercise Trainings[Title/Abstract])) OR (Training, Exercise[Title/Abstract])) OR (Trainings, Exercise[Title/Abstract])) OR (Exercise Therapy[MeSH Terms])) OR (Rehabilitation Exercise[Title/Abstract])) OR (Exercise, Rehabilitation[Title/Abstract])) OR (Exercises, Rehabilitation[Title/Abstract])) OR (Rehabilitation Exercises[Title/Abstract])) OR (Therapy, Exercise[Title/Abstract])) OR (Exercise Therapies[Title/Abstract])) OR (Therapies, Exercise[Title/Abstract])) OR (Remedial Exercise[Title/Abstract])) OR (Exercise, Remedial[Title/Abstract])) OR (Exercises, Remedial[Title/Abstract])) OR (Remedial Exercises[Title/Abstract])) OR (Resistance Training[MeSH Terms])) OR (Training, Resistance[Title/Abstract])) OR (Strength Training[Title/Abstract])) OR (Training, Strength[Title/Abstract])) OR (Weight-Lifting Strengthening Program[Title/Abstract])) OR (Strengthening Programs, Weight-Lifting[Title/Abstract])) OR (Weight Lifting Strengthening Program[Title/Abstract])) OR (Weight-Lifting Exercise Program[Title/Abstract])) OR (Exercise Programs, Weight-Lifting[Title/Abstract])) OR (Weight Lifting Exercise Program[Title/Abstract])) OR (Strengthening Programs, Weight-Bearing[Title/Abstract])) OR (Endurance Training[MeSH Terms])) OR (High-Intensity Interval Training[MeSH Terms])) OR (Circuit-Based Exercise[MeSH Terms])) OR (taichi[Title/Abstract])) OR (yoga[Title/Abstract])) OR (pilates[Title/Abstract])) OR (qi gong[Title/Abstract])) OR (baduanjin[Title/Abstract])) OR (walking[Title/Abstract])  #2. ((((((((((((cancer[Title/Abstract]) OR (Colorectal cancer[Title/Abstract])) OR (tumor[Title/Abstract])) OR (oncology[Title/Abstract])) OR (chemotherapy[Title/Abstract])) OR (radiotherapy[Title/Abstract])) OR (Cancer Survivors[MeSH Terms])) OR (Cancer Survivor[Title/Abstract])) OR (Survivors, Cancer[Title/Abstract])) ) OR (Long-Term Cancer Survivor[Title/Abstract])) OR (Long Term Cancer Survivors[Title/Abstract])) OR (Cancer Survivorship[Title/Abstract])  #3. (((((((((fatigue[MeSH Terms]) OR (healthy[Title/Abstract])) OR (Quality of Life[Title/Abstract])) OR (Biomarkers[Title/Abstract])) OR (Physical functions[Title/Abstract])) OR (Athletic ability[Title/Abstract])) OR (Cardiopulmonary function[Title/Abstract])) OR (Muscle Strength[Title/Abstract])) OR (Cytokines[Title/Abstract])) OR (BMI[Title/Abstract])  #4. ((randomized controlled trial[Publication Type]) OR (randomized[Title/Abstract])) OR (placebo[Title/Abstract])  #5. #1 AND #2 AND #3 AND #4 |
| Embase | #1. ('exercise'/exp OR 'physical exercise':ti,ab OR 'physical exercises':ti,ab OR 'physical activity':ti,ab OR 'activities, physical':ti,ab OR 'activity, physical':ti,ab OR 'physical activities':ti,ab OR 'aerobic exercise':ti,ab OR 'isometric exercise':ti,ab OR 'acute exercise':ti,ab OR 'exercise training':ti,ab OR 'rehabilitation exercise':ti,ab OR 'exercise therapy'/exp OR 'resistance training'/exp OR 'strength training':ti,ab OR 'weight lifting strengthening program':ti,ab OR 'endurance training'/exp OR 'high intensity interval training'/exp OR 'circuit-based exercise'/exp OR 'taichi':ti,ab OR 'yoga':ti,ab OR 'pilates':ti,ab OR 'qi gong':ti,ab OR 'baduanjin':ti,ab OR 'walking':ti,ab)  #2. ('colorectal cancer'/exp OR 'colorectal cancer':ti,ab OR 'colon cancer':ti,ab OR 'rectal cancer':ti,ab OR 'colorectal tumor':ti,ab OR 'colorectal neoplasm':ti,ab OR 'colorectal carcinoma':ti,ab OR 'colon neoplasm':ti,ab OR 'rectal neoplasm':ti,ab OR 'colon carcinoma':ti,ab OR 'rectal carcinoma':ti,ab OR 'colorectal adenocarcinoma':ti,ab OR 'colorectal oncology':ti,ab OR 'colorectal cancer survivor'/exp OR 'long-term colorectal cancer survivor':ti,ab OR 'colorectal cancer survivorship':ti,ab OR 'chemotherapy':ti,ab OR 'radiotherapy':ti,ab)  #3. (('fatigue'/exp OR 'tiredness':ti,ab OR 'physical fatigue':ti,ab OR 'mental fatigue':ti,ab OR 'chronic fatigue syndrome'/exp OR 'fatigue severity scale':ti,ab OR 'fatigue resistance':ti,ab) OR ('healthy'/exp OR 'health status':ti,ab OR 'physical health':ti,ab OR 'well-being'/exp OR 'mental health':ti,ab OR 'general health':ti,ab OR 'health-related quality of life':ti,ab OR 'self-rated health':ti,ab OR 'health survey'/exp OR 'sf-36':ti,ab OR 'sf-12':ti,ab OR 'sf-8':ti,ab OR 'hrqol':ti,ab)OR ('physical function'/exp OR 'physical fitness'/exp OR 'physical performance':ti,ab OR 'physical ability':ti,ab OR 'functional ability':ti,ab OR 'functional capacity':ti,ab OR 'functional performance':ti,ab OR 'daily living activities'/exp OR 'adl':ti,ab OR 'physical endurance':ti,ab OR 'functional decline':ti,ab OR 'disability'/exp OR 'physical independence':ti,ab)OR ('athletic ability':ti,ab OR 'physical aptitude':ti,ab OR 'sports performance':ti,ab OR 'endurance':ti,ab OR 'exercise capacity':ti,ab OR 'exercise performance':ti,ab OR 'cardiorespiratory fitness'/exp OR 'aerobic capacity':ti,ab OR 'muscle performance':ti,ab OR 'strength performance':ti,ab) OR ('biomarker'/exp OR 'biological marker':ti,ab OR 'inflammatory marker':ti,ab OR 'cytokine'/exp OR 'c-reactive protein'/exp OR 'tumor necrosis factor alpha':ti,ab OR 'interleukin-6':ti,ab OR 'interleukin-10':ti,ab OR 'biomarker panel':ti,ab OR 'metabolic markers':ti,ab OR 'oxidative stress markers':ti,ab OR 'hormonal biomarkers':ti,ab OR 'genetic marker'/exp OR 'immune markers':ti,ab) OR ('muscle strength'/exp OR 'muscle power':ti,ab OR 'muscle endurance':ti,ab OR 'grip strength'/exp OR 'isometric strength':ti,ab OR 'dynamic strength':ti,ab OR 'leg strength':ti,ab OR 'upper body strength':ti,ab OR 'lower body strength':ti,ab OR 'muscle function'/exp))  #4. (('randomized controlled trial'/exp OR 'randomized controlled trials':ti,ab OR 'randomized trial':ti,ab OR 'controlled clinical trial'/exp OR 'controlled trial':ti,ab OR 'random allocation':ti,ab OR 'double-blind method'/exp OR 'single-blind method'/exp OR 'placebo'/exp OR 'placebo-controlled':ti,ab OR 'randomized':ti,ab OR 'clinical trial':ti,ab OR 'trial protocol':ti,ab OR 'double blind':ti,ab OR 'single blind':ti,ab OR 'crossover trial'/exp OR 'parallel group':ti,ab OR 'random assignment':ti,ab OR 'multicenter study'/exp OR 'multicenter trial':ti,ab OR 'randomization'/exp OR 'randomized study':ti,ab OR 'randomised':ti,ab OR 'randomised controlled trial':ti,ab OR 'sham control':ti,ab OR 'placebo effect':ti,ab OR 'placebo response':ti,ab OR 'control group'/exp OR 'trial design'/exp OR 'intervention study'/exp))  #5. #1 AND #2 AND #3 AND #4 |
| Web of Science | #1. TS=("exercise" OR "physical exercise" OR "physical exercises" OR "physical activity" OR "activities, physical" OR "activity, physical" OR "physical activities" OR "aerobic exercise" OR "isometric exercise" OR "acute exercise" OR "exercise training" OR "rehabilitation exercise" OR "exercise therapy" OR "resistance training" OR "strength training" OR "weight lifting strengthening program" OR "endurance training" OR "high intensity interval training" OR "circuit-based exercise" OR "taichi" OR "yoga" OR "pilates" OR "qi gong" OR "baduanjin" OR "walking")  #2. TS=("colorectal cancer" OR "colon cancer" OR "rectal cancer" OR "colorectal neoplasm" OR "colorectal carcinoma" OR "colon neoplasm" OR "rectal neoplasm" OR "colon carcinoma" OR "rectal carcinoma" OR "colorectal adenocarcinoma" OR "colorectal tumor" OR "colorectal malignancy" OR "colorectal oncology")  #3. TS=(("fatigue" OR "tiredness" OR "physical fatigue" OR "mental fatigue" OR "chronic fatigue syndrome" OR "fatigue severity scale" OR "fatigue resistance") OR ("healthy" OR "health status" OR "physical health" OR "well-being" OR "mental health" OR "general health" OR "health-related quality of life" OR "self-rated health" OR "health survey" OR "sf-36" OR "sf-12" OR "sf-8" OR "hrqol") OR ("physical function" OR "physical fitness" OR "physical performance" OR "physical ability" OR "functional ability" OR "functional capacity" OR "functional performance" OR "activities of daily living" OR "adl" OR "physical endurance" OR "functional decline" OR "disability" OR "physical independence") OR ("athletic ability" OR "physical aptitude" OR "sports performance" OR "endurance" OR "exercise capacity" OR "exercise performance" OR "cardiorespiratory fitness" OR "aerobic capacity" OR "muscle performance" OR "strength performance") OR ("biomarker" OR "biological marker" OR "inflammatory marker" OR "cytokine" OR "c-reactive protein" OR "tumor necrosis factor alpha" OR "interleukin-6" OR "interleukin-10" OR "biomarker panel" OR "metabolic markers" OR "oxidative stress markers" OR "hormonal biomarkers" OR "genetic marker" OR "immune markers") OR ("muscle strength" OR "muscle power" OR "muscle endurance" OR "grip strength" OR "isometric strength" OR "dynamic strength" OR "leg strength" OR "upper body strength" OR "lower body strength" OR "muscle function"))  #4. TS=("randomized controlled trial" OR "randomized controlled trials" OR "randomized trial" OR "controlled clinical trial" OR "controlled trial" OR "random allocation" OR "double-blind method" OR "single-blind method" OR "placebo" OR "placebo-controlled" OR "randomized" OR "clinical trial" OR "trial protocol" OR "double blind" OR "single blind" OR "crossover trial" OR "parallel group" OR "random assignment" OR "multicenter study" OR "multicenter trial" OR "randomization" OR "randomized study" OR "randomised" OR "randomised controlled trial" OR "sham control" OR "placebo effect" OR "placebo response" OR "control group" OR "trial design" OR "intervention study")  #5. #1 AND #2 AND #3 AND #4 |
| Cochrane library | #1. ("exercise" OR "physical exercise" OR "physical exercises" OR "physical activity" OR "activities, physical" OR "activity, physical" OR "physical activities" OR "aerobic exercise" OR "isometric exercise" OR "acute exercise" OR "exercise training" OR "rehabilitation exercise" OR "exercise therapy" OR "resistance training" OR "strength training" OR "weight lifting strengthening program" OR "endurance training" OR "high intensity interval training" OR "circuit-based exercise" OR "taichi" OR "yoga" OR "pilates" OR "qi gong" OR "baduanjin" OR "walking")  #2. ("colorectal cancer" OR "colon cancer" OR "rectal cancer" OR "colorectal neoplasm" OR "colorectal carcinoma" OR "colon neoplasm" OR "rectal neoplasm" OR "colon carcinoma" OR "rectal carcinoma" OR "colorectal adenocarcinoma" OR "colorectal tumor" OR "colorectal malignancy" OR "colorectal oncology" OR "colorectal cancer survivor" OR "long-term colorectal cancer survivor" OR "colorectal cancer survivorship" OR "chemotherapy" OR "radiotherapy")  #3. (("fatigue" OR "tiredness" OR "physical fatigue" OR "mental fatigue" OR "chronic fatigue syndrome" OR "fatigue severity scale" OR "fatigue resistance")OR ("healthy" OR "health status" OR "physical health" OR "well-being" OR "mental health" OR "general health" OR "health-related quality of life" OR "self-rated health" OR "health survey" OR "sf-36" OR "sf-12" OR "sf-8" OR "hrqol")OR ("physical function" OR "physical fitness" OR "physical performance" OR "physical ability" OR "functional ability" OR "functional capacity" OR "functional performance" OR "daily living activities" OR "adl" OR "physical endurance" OR "functional decline" OR "disability" OR "physical independence")OR ("athletic ability" OR "physical aptitude" OR "sports performance" OR "endurance" OR "exercise capacity" OR "exercise performance" OR "cardiorespiratory fitness" OR "aerobic capacity" OR "muscle performance" OR "strength performance") OR ("biomarker" OR "biological marker" OR "inflammatory marker" OR "cytokine" OR "c-reactive protein" OR "tumor necrosis factor alpha" OR "interleukin-6" OR "interleukin-10" OR "biomarker panel" OR "metabolic markers" OR "oxidative stress markers" OR "hormonal biomarkers" OR "genetic marker" OR "immune markers") OR ("muscle strength" OR "muscle power" OR "muscle endurance" OR "grip strength" OR "isometric strength" OR "dynamic strength" OR "leg strength" OR "upper body strength" OR "lower body strength" OR "muscle function"))  #4. ("randomized controlled trial" OR "randomized controlled trials" OR "randomized trial" OR "controlled clinical trial" OR "controlled trial" OR "random allocation" OR "double-blind method" OR "single-blind method" OR "placebo" OR "placebo-controlled" OR "randomized" OR "clinical trial" OR "trial protocol" OR "double blind" OR "single blind" OR "crossover trial" OR "parallel group" OR "random assignment" OR "multicenter study" OR "multicenter trial" OR "randomization" OR "randomized study" OR "randomised" OR "sham control" OR "placebo effect" OR "placebo response" OR "control group" OR "trial design" OR "intervention study")  #5. #1 AND #2 AND #3 AND #4 |
| EBSCOhost | #1. (MH "Exercise" OR TI "physical exercise" OR AB "physical exercise" OR TI "physical exercises" OR AB "physical exercises" OR TI "physical activity" OR AB "physical activity" OR TI "activities, physical" OR AB "activities, physical" OR TI "aerobic exercise" OR AB "aerobic exercise" OR TI "isometric exercise" OR AB "isometric exercise" OR TI "acute exercise" OR AB "acute exercise" OR TI "exercise training" OR AB "exercise training" OR TI "rehabilitation exercise" OR AB "rehabilitation exercise" OR (MH "Exercise Therapy") OR (MH "Resistance Training") OR TI "strength training" OR AB "strength training" OR TI "weight lifting strengthening program" OR AB "weight lifting strengthening program" OR (MH "Endurance Training") OR (MH "High Intensity Interval Training") OR (MH "Circuit Training") OR TI "taichi" OR AB "taichi" OR TI "yoga" OR AB "yoga" OR TI "pilates" OR AB "pilates" OR TI "qi gong" OR AB "qi gong" OR TI "baduanjin" OR AB "baduanjin" OR TI "walking" OR AB "walking")  #2. (MH "Neoplasms" OR TI "cancer" OR AB "cancer" OR TI "colorectal cancer" OR AB "colorectal cancer" OR TI "tumor" OR AB "tumor" OR TI "oncology" OR AB "oncology" OR TI "chemotherapy" OR AB "chemotherapy" OR TI "radiotherapy" OR AB "radiotherapy" OR (MH "Cancer Survivors") OR TI "long-term cancer survivor" OR AB "long-term cancer survivor" OR TI "cancer survivorship" OR AB "cancer survivorship")  #3. ((MH "Fatigue" OR TI "fatigue" OR AB "fatigue" OR TI "tiredness" OR AB "tiredness" OR TI "physical fatigue" OR AB "physical fatigue" OR TI "mental fatigue" OR AB "mental fatigue" OR (MH "Chronic Fatigue Syndrome") OR TI "fatigue severity scale" OR AB "fatigue severity scale" OR TI "fatigue resistance" OR AB "fatigue resistance")OR (MH "Health Status" OR TI "healthy" OR AB "healthy" OR TI "health status" OR AB "health status" OR TI "well-being" OR AB "well-being" OR (MH "Mental Health") OR TI "general health" OR AB "general health" OR TI "health-related quality of life" OR AB "health-related quality of life" OR TI "self-rated health" OR AB "self-rated health" OR (MH "Health Surveys") OR TI "SF-36" OR AB "SF-36" OR TI "SF-12" OR AB "SF-12" OR TI "SF-8" OR AB "SF-8" OR TI "HRQoL" OR AB "HRQoL")OR (MH "Physical Fitness" OR TI "physical fitness" OR AB "physical fitness" OR TI "physical performance" OR AB "physical performance" OR TI "physical ability" OR AB "physical ability" OR TI "functional ability" OR AB "functional ability" OR TI "functional capacity" OR AB "functional capacity" OR TI "activities of daily living" OR AB "activities of daily living" OR (MH "Activities of Daily Living") OR TI "physical endurance" OR AB "physical endurance" OR TI "functional decline" OR AB "functional decline" OR (MH "Disability") OR TI "physical independence" OR AB "physical independence"))  #4. (MH "Randomized Controlled Trials" OR TI "randomized controlled trials" OR AB "randomized controlled trials" OR TI "randomized trial" OR AB "randomized trial" OR (MH "Controlled Clinical Trials") OR TI "controlled trial" OR AB "controlled trial" OR TI "random allocation" OR AB "random allocation" OR (MH "Double-Blind Method") OR (MH "Single-Blind Method") OR (MH "Placebos") OR TI "placebo" OR AB "placebo" OR TI "randomized" OR AB "randomized" OR TI "clinical trial" OR AB "clinical trial" OR TI "trial protocol" OR AB "trial protocol" OR TI "double blind" OR AB "double blind" OR TI "single blind" OR AB "single blind" OR (MH "Crossover Design") OR TI "parallel group" OR AB "parallel group" OR TI "random assignment" OR AB "random assignment" OR (MH "Multicenter Studies") OR TI "multicenter trial" OR AB "multicenter trial" OR TI "randomization" OR AB "randomization" OR TI "randomised" OR AB "randomised" OR TI "sham control" OR AB "sham control" OR TI "placebo effect" OR AB "placebo effect" OR TI "control group" OR AB "control group" OR (MH "Trial Design") OR (MH "Intervention Studies"))  #5. #1 AND #2 AND #3 AND #4 |

**Supplementary information 3. Definitions and units of included health outcomes.**

| ID | Outcome measure | Unit |
| --- | --- | --- |
| A | Body Composition (BC) |  |
| A1* | BMI (Body mass index) | kg/m^2^ |
| A2 | Total lean mass | kg |
| A3 | Lean body mass | % |
| A4* | Total fat mass | kg |
| A5 | Appendicular lean mass | kg |
| A6 | Lean-to-fat ratio | % |
| A7* | Visceral adipose tissue | cm^2^ |
| A8* | Subcutaneous adipose tissue | cm^2^ |
| A9 | Bone mineral density | g/cm^2^ |
| A10* | Weight/Body mass | kg |
| A11* | Waist to hip ratio | % |
| A12* | Sagittal abdominal diameter | cm |
| A13 | Waist circumference | cm |
| A14 | Hip circumference | cm |
| A15 | MFR (muscle-to-fat ratio) | % |
| A16* | Body fat | % |
| A17* | Sum of skinfolds | mm |
| A18 | Muscle structure (Thickness external oblique) | cm |
| A19 | Muscle structure (Thickness internal oblique) | cm |
| A20 | Muscle structure (Thickness transversus abdominalis) | cm |
| A21 | Muscle structure (Depth lumbar multifidus) | cm |
| B | Cancer-Associated Biomarkers (CAB) |  |
| B1* | CRP (C-reactive protein) | mg/L |
| B2* | hs-CRP (high-sensitivity C-reactive protein) | mg/dl, mg/L |
| B3* | IL-6 (Interleukin-6) | pg/ml |
| B4* | TNF-α (Tumor necrosis factor-alpha) | pg/ml |
| B5* | sTNFαR2 (Soluble tumor necrosis factor receptor 2) | pg/ml |
| B6* | sICAM-1 (Soluble intercellular adhesion molecule-1) | ng/ml |
| B7* | sVCAM-1 (Soluble vascular cell adhesion molecule-1) | ng/ml |
| B8* | Insulin | mg/dl, pmol/L, μU/ml |
| B9* | Glucose | mg/dl, mmol/L |
| B10* | Insulin resistance | HOMA-IR |
| B11* | IGF-1 (Insulin-like growth factor-1) | nmol/L |
| B12* | C-peptide | nmol/L |
| B13* | Fructosamine | nmol/L |
| B14* | NLR (Neutrophil-to-lymphocyte ratio) | % |
| B15 | IGFBP-3 (Insulin-like growth factor binding protein-3) | nmol/L |
| B16* | CTC count (Circulating tumor cell count) | per 1 ml blood |
| B17* | Total cholesterol | mg/dl, mM |
| B18* | Triglycerides | mg/dl, mM |
| B19 | HDL (High-density lipoprotein) cholesterol | mg/dl, mM |
| B20* | LDL (Low-density lipoprotein) cholesterol | mM |
| B21* | Leptin | pg/ml |
| B22 | Adiponectin | μg/ml |
| B23 | Hemoglobin | mmol/L |
| B24* | Creatinine | µmol/L |
| B25 | Albumin | g/L |
| C | Cardiorespiratory Fitness (CRF) |  |
| C1 | VO_2_ at AT (VO_2_ at the anaerobic threshold) | ml·kg^-1^·min^-1^ |
| C2 | VO_2_ at VT (VO_2_ at the ventilatory threshold) | L/min |
| C3 | Power output at VT | Watt |
| C4 | VO_2peak_ | ml·kg^-1^·min^-1^ |
| C5 | VO_2peak_ | L/min |
| C6 | VO_2max_ | ml·kg^-1^·min^-1^ |
| C7 | Watt_max_ | watt |
| C8 | HR_max_ | bpm |
| C9 | VO_2_ pulse peak | ml/beat |
| C10* | Step test | bpm |
| C11 | Ergospirometry | Efficiency |
| C12 | Exercise tolerance | s |
| D | Muscular Fitness (MF) |  |
| D1 | Handgrip strength | kg |
| D2 | h1RM (bench press) | kg |
| D3 | h1RM (leg press) | kg |
| D4 | h1RM (lat pulldown) | kg |
| D5 | core muscle strength | mmHg |
| D6 | Push up | reps |
| D7 | Back muscle strength | kg |
| D8 | MVT (Maximum Voluntary Torque) | Nm |
| D9 | 6-Minute Walk Distance | m |
| D10 | Core muscle endurance | s |
| D11* | Treadwalk | min |
| D12* | Delta sEMG | μV |
| E | Physical Function Capacity (PFC) |  |
| E1 | 30-s Sit-to-stand | reps |
| E2* | 5-reps Sit-to-stand | s |
| E3 | Timed up and go | s |
| E4* | Stair climb test | s |
| E5* | 4-m gait time | s |
| E6* | 8-foot up and go | s |
| E7 | Gleichgewichtstest(balance test) | score |
| E8 | Sit-to-stand mean power | W |
| E9 | Relative sit-to-stand mean power | W/kg |
| E10 | Short physical performance battery | score |
| E11 | Sit-to-stand | score |
| E12 | Balance | score |
| E13 | Gait speed score | score |
| F | Health-Related Quality of Life (HRQoL) |  |
| F1 | FACT-C (Functional Assessment of Cancer Therapy - Colorectal) | score |
| F2 | FACT-PWB (Physical Well-Being) | score |
| F3 | FACT-FWB (Functional Well-Being) | score |
| F4 | FACT-NTX (Neurotoxicity Subscale) | score |
| F5 | FACT-SocWB (Social/Family Well-Being) | score |
| F6 | FACT-EmoWB (Emotional Well-Being) | score |
| F7 | FACT-G (Functional Assessment of Cancer Therapy – General) | score |
| F8 | EORTC QLQ-C30 (European Organisation for Research and Treatment of Cancer) | score |
| F9 | EORTC (QoL) | score |
| F10 | EORTC (Physical functioning) | score |
| F11 | EORTC (Role functioning) | score |
| F12 | EORTC (Emotional functioning) | score |
| F13 | EORTC (Cognitive functioning) | score |
| F14 | EORTC (Social functioning) | score |
| F15* | EORTC (Fatigue) | score |
| F16* | EORTC (Pain) | score |
| F17 | SF-36 (Physical function subscale Short Form–36) | score |
| F18 | SF-36 (Medical Outcomes Survey Short Form) | score |
| F19 | SF-36 (Medical Outcomes Survey Short Form) | score |
| F20 | SF-36 (Physical functioning) | score |
| F21 | SF-36 (Role physical) | score |
| F22 | SF-36 (bodily pain) | score |
| F23 | SF-36 (general health) | score |
| F24 | SF-36 (vitality) | score |
| F25 | SF-36 (social functioning) | score |
| F26 | SF-36 (role emotional) | score |
| F27 | SF-36 (mental health) | score |
| F28 | SWLS (Satisfaction with Life Scale) | score |
| F29* | FCRI (Fear of Cancer Recurrence Inventory) | score |
| F30 | BARSE (Barrier-Specific Self-Efficacy Scale) | score |
| F31 | Positive affect score | score |
| F32* | Negative affect score | score |
| F33* | GAD-7 (Generalized Anxiety Disorder-7) | score |
| F34* | HADS (Hospital Anxiety and Depression Scale) Anxiety | score |
| F35* | STAI (State-Trait Anxiety Inventory) | score |
| F36* | PHQ-9 (Patient Health Questionnaire-9) | score |
| F37* | HADS (Hospital Anxiety and Depression Scale) Depression | score |
| F38* | Depression (0-60) | score |
| F39* | BDI-II (Beck Depression Inventory-II) | score |
| F40* | FSI (Fatigue Symptom Inventory) | score |
| F41 | FACIT-FS (Functional Assessment of Chronic Illness Therapy-Fatigue Scale) | score |
| F42* | MFI-20 (Multidimensional Fatigue Inventory-20) | score |
| F43* | FQL (Fatigue Quality of Life Scale) | score |
| F44 | FACT-F (Functional Assessment of Chronic Illness Therapy-Fatigue Scale) | score |
| F45* | PSQI (Pittsburgh Sleep Quality Index) | score |
| F46* | HADS (Hospital Anxiety and Depression Scale) Depression and Anxiety | score |

Note: The effect size marked with an asterisk (*) was originally negative to indicate favorable intervention outcomes; after conversion, positive values now represent favorable intervention outcomes.

**Supplementary information 4. Information on RCTs Included in the Meta-Analysis.**

| Study | Study registration number | Intervening measure and intensity | Interventions duration (minutes per session, times per week, total weeks) | Inflammatory outcome |
| --- | --- | --- | --- | --- |
| Caan2024, Brown2024[1, 2] | NCT03291951 | RT: 5 large muscle exercises (eg, bench press, squats, 1-arm row, lunges, and deadlifts);  65-85% 1RM, 3-5 sets, 6-10 reps/set. | 2 times/week,  18 weeks. | A1-2, A5-6; D1; E2, E5, E8, E9, E10-13; F17. |
| TrigueroCánovas2023[3] | NCT03618329 | AE+RT: walk, 1/2/3 week, 30/40/50 min/day, 3500/5000/7500 step/day;  Bodyweight muscle endurance training, 20 min. | AE everyday,  RT 3 times/week,  8 weeks. | C11; D9. |
| Molenaar2023[4] | NTR5947 | AE+RT: bicycle, HIIT 2 min/sets, 4 set, 85-90% peak power;  2 large muscle exercises, 65-70% 1RM, 10 reps/set. | 3 times/week,  4 weeks. | C1, C4; D1-2, D9; F8, F33, F36. |
| Leach2023[5] | IRB#18-2436 | AE+RT: ZOOM live sports classes, 2 types of AE and 2 types of RT;  40-70% HRR or 11-14 RPE, 30-45 min/time. | 4 times/week,  12 weeks. | C6; D2-3, D6; E1; F30. |
| Brown2023, 2018CCC, 2018EC, 2018P, 2018PO,2017[6-11] | NCT02250053 | AE: Treadmill, 50-70% HR_max_, 2 intervention groups, 150/300 min/week. | 24 weeks. | A1-2, A4, A7-14; B2-3, B5-16; F1, F18-19, F29, F40, F45. |
| Bojesen2023[12] | NCT04167436 | AE+RT: bicycle, HIIT 2 min/sets, 4 set, 90% HR_max_;  2 types of RT , 65-70% 1RM, 3 sets, 8-12 reps/set. | 3 times/week,  4 weeks. | B1, B14, B23-25; C1, C4; D1, D9; E1. |
| Hwang2022[13] | KCT0004796 | RT: 6 types of RT, 20 min;  6 types of functional training, 10 min;  1-2/3-4/5-6 week, 1-2/2-3/3 sets, 10 reps/set. | everyday,  6 weeks. | A1-2, A10, A13; B2, B8-9, B17-19; D1, D5, D10; E1. |
| Waller2020[14] | NCT04047524 | AE+RT: RT 2 sets, 8-10 reps/set;  50-70% HR_max_ or 11-14 Borg scale. | AE 5 times/week,  RT 2 times/week,  4 weeks. | D9; F34, F37, F46. |
| Northgraves2020[15] | NCT02264496 | AE+RT: bicycle;  Core Strength Exercises;  40-60% HRR or 11-13 Borg scale. | 3 times/week,  4 weeks. | D1, D9; E2-4. |
| Shim2019[16] | KUGH16034-001 | AE+RT: 6 types of AE and 6 types of RT;  1 min/type, 30 min/time. | 3 times/week,  24 weeks. | A15; B21-22. |
| Moug2019[17] | ISRCTN62859294 | PA: wale, 1-2/3-4/5-6/7-8/9-17 week, 3/5/3/5 day/week, add 1500/1500/3000/3000 steps/day. | 12 weeks. | A1, A10, A13; D9; E1; F1, F15, F31-32, F39 |
| Kim2019[18] | - | AE+RT: 5 types of AE and 5 types of RT; 3 sets, 12-15 reps/set. | everyday,  12 weeks. | F1, F36, F41 |
| Gillis2019[19] | NCT02586701,  NCT01356264 | AE+RT: walk/cycle/jog 30 min;  8 types of RT 25 min, 2 sets, 8-15 reps/set;  60-70% HR_max._ | 4 times/week,  4 weeks. | A3, A10. |
| Christensen2019[20] | NCT02403024 | AE: cycle of 3 minutes slow walking and 3 minutes brisk walking. | 150 min/week,  12 weeks. | B3-4, B17-21; C4; F1. |
| Zimmer2018[21] | DRKS00011719 | AE+RT: AE 10 min, balance and coordination training 15 min, 60-70% HR_max_ or 12-13 Borg scale;  5 types of RT, 60-80% 1RM. | 2 times/week,  8 weeks. | D2-4, D9; E7; F2-7. |
| Lee2018[22] | - | AE+RT: brisk walking/ hiking/stationary biking;  7 types of core and RT, 3 sets, 15 reps/set. | 5 times/week,  6 weeks. | A1 ,A10, A13, A16; C10; D6, D9; E1. |
| BousquetDion2018[23] | NCT02586701 | AE+RT: walk/cycle/jog 30 min;  8 types of RT 25 min, 2 sets, 8-15 reps/set;  60-70% HR_max._ | 4 times/week,  4 weeks. | D9. |
| Lee2017[24] | ISRCTN47234641 | AE+RT: brisk walking/ hiking/stationary biking;  Bodyweight muscle endurance training, 30 min;  65% HRR. | everyday,  12 weeks. | A1-2, A10, A13, A16; B2, B4, B8-10, B17-19, B22; D1, D6, D9; E1. |
| CantareroVillanueva2016,2017[25, 26] | NCT02052050 | AE+RT: running/fast walking 25 min;  lumbar and pelvic stabilization exercises, 1/3 sets, 8-10/8 reps/set;  6-20 Borg scale. | 3 times/week,  8 weeks. | A1-2, A10, A13-14, A16, A18-21; D7, D9-10. |
| VanVulpen2016[27] | ISRCTN43801571 | AE+RT: MIIT with HR at (3*2 min increasing to 2*7 min) and below (3*4 min decreasing to 1*7 min) ventilatory threshold;  Muscle strength training was performed for all major muscle groups, 2/1/1 sets, 10/10/20 reps/set, 65%/75%/45% 1 RM;  MIIT and RT, 40min. | 2 times/week,  18 weeks. | A10; C2-5, C7-8; F9-16, F34, F37, F42-43. |
| Courneya2016[28] | NCT00819208 | PA: increase by at least 10 MET-hours/week. | everyday,  48 weeks. | A10, A13-14; C6; D6, D9; E1, E6. |
| Gillis2014[29] | NCT01356264 | AE+RT: walk/cycle/jog 20 min;  8 types of RT 20 min, 2 sets, 8-15 reps/set;  40%+ HRR or 12+ Borg scale. | 3 times/week,  4 weeks. | D9, F20-27, F34, F37. |
| Pinto2013[30] | - | AE: walk/cycle/jog, 1/12 week, 2/5 day/week, 10/30 min/day, 64-76 HR_max_. | 12 weeks. | C4; D11; F1, F17, F41. |
| Bourke 2011[31] | - | AE+RT: treadmills/rowing/bicycle, 30min;  large muscle group training, 2-4 sets, 8-12 reps/set;  55-85% HRR or 11-13 Borg scale. | 3 times/week,  12 weeks. | A1, A10-11; C12; D8, D12; E1; F1. |
| Kim2009[32] | - | AE: 1/2/3-4 week, 20/30/30 min, 40/50/60-65% HRR. | everyday,  4 weeks. | C3, C5-6, C8-9. |
| Courneya2003[33] | - | AE: 20-30 min, 65-75% HR_max_. | 3-5 times/week,  16 weeks. | A1, A17; C12; F1, F28, F35, F38, F44. |

**Supplementary information 5.Overall effect and sensitivity analyses**

Table S1. RVE model parameters without covariates

| Outcome domain | ES | SE | t | df | p | CI.L | CI.U | sig | I² | V mean |
| --- | --- | --- | --- | --- | --- | --- | --- | --- | --- | --- |
| Body Composition (BC) | 0.055 | 0.021 | 2.608 | 7.098 | 0.035 | 0.005 | 0.104 | ** | 0 | 0.1317 |
| Cancer-Associated Biomarkers (CAB) | 0.162 | 0.094 | 1.725 | 3.177 | 0.178 | -0.127 | 0.451 |  | 0 | 0.1764 |
| Cardiorespiratory Fitness (CRF) | 0.354 | 0.058 | 6.079 | 6.468 | 0.001 | 0.214 | 0.494 | *** | 5.74% | 0.0076 |
| Muscular Fitness (MF) | 0.447 | 0.104 | 4.305 | 15.147 | 0.001 | 0.226 | 0.668 | *** | 47.20% | 0.1434 |
| Physical function capacity (PFC) | 0.216 | 0.148 | 1.464 | 8.509 | 0.179 | -0.121 | 0.554 |  | 55.19% | 0.1327 |
| Health-Related Quality of Life (HRQoL) | 0.032 | 0.062 | 0.524 | 10.733 | 0.611 | -0.104 | 0.169 |  | 32.09% | 0.1065 |
| Overall | 0.198 | 0.032 | 6.213 | 21.439 | 0.000 | 0.132 | 0.264 | *** | 30.1% | 0.1358 |

Note: ES, effect size; SE, standard error; df, degrees of freedom; CI, confidence interval; RVE, robust variance estimation.

Table S2. Sensitivity of pooled effect estimates to the assumed within-study correlation (ρ)

| No. | ρ | BC | CAB | CRF | MF | PFC | HRQoL | Overall |
| --- | --- | --- | --- | --- | --- | --- | --- | --- |
| 1 | 0.1 | 0.055 | 0.162 | 0.354 | 0.447 | 0.216 | 0.032 | 0.198 |
| 2 | 0.15 | 0.055 | 0.162 | 0.354 | 0.447 | 0.216 | 0.032 | 0.198 |
| 3 | 0.2 | 0.055 | 0.162 | 0.354 | 0.447 | 0.216 | 0.032 | 0.198 |
| 4 | 0.25 | 0.055 | 0.162 | 0.354 | 0.447 | 0.216 | 0.032 | 0.198 |
| 5 | 0.3 | 0.055 | 0.162 | 0.354 | 0.447 | 0.216 | 0.032 | 0.198 |
| 6 | 0.35 | 0.055 | 0.162 | 0.354 | 0.447 | 0.216 | 0.032 | 0.198 |
| 7 | 0.4 | 0.055 | 0.162 | 0.354 | 0.447 | 0.216 | 0.032 | 0.198 |
| 8 | 0.45 | 0.055 | 0.162 | 0.354 | 0.447 | 0.216 | 0.032 | 0.198 |
| 9 | 0.5 | 0.055 | 0.162 | 0.354 | 0.447 | 0.216 | 0.032 | 0.198 |
| 10 | 0.55 | 0.055 | 0.162 | 0.354 | 0.447 | 0.216 | 0.032 | 0.198 |
| 11 | 0.6 | 0.055 | 0.162 | 0.354 | 0.447 | 0.216 | 0.032 | 0.198 |
| 12 | 0.65 | 0.055 | 0.162 | 0.354 | 0.447 | 0.216 | 0.032 | 0.198 |
| 13 | 0.7 | 0.055 | 0.162 | 0.354 | 0.447 | 0.216 | 0.032 | 0.198 |
| 14 | 0.75 | 0.055 | 0.162 | 0.354 | 0.447 | 0.217 | 0.032 | 0.198 |
| 15 | 0.8 | 0.055 | 0.162 | 0.354 | 0.447 | 0.217 | 0.032 | 0.198 |
| 16 | 0.85 | 0.055 | 0.162 | 0.354 | 0.447 | 0.217 | 0.032 | 0.198 |
| 17 | 0.9 | 0.055 | 0.163 | 0.354 | 0.447 | 0.217 | 0.032 | 0.198 |

Note: Values are pooled effect estimates from RVE models fitted using different assumed within-study correlations (ρ).

**Supplementary information 6. Risk of Bias and Publication Bias.**

Note: D1, bias arising from the randomization process; D2, bias due to deviations from intended interventions; D3, bias due to missing outcome data; D4, bias in measurement of the outcome; D5, bias in selection of the reported result. Green indicates low risk of bias, yellow indicates some concerns, and red indicates high risk of bias.

Fig. S1. Summary of risk of bias across included studies

Fig. S2. Risk of bias assessment for each included study

Table S3. Summary of risk of bias judgments and supporting rationale for each included study

| **Study** | **D1** | **Reason** | **D2** | **Reason** | **D3** | **Reason** | **D4** | **Reason** | **D5** | **Reason** | **Overall** | **Overall rationale** |
| --- | --- | --- | --- | --- | --- | --- | --- | --- | --- | --- | --- | --- |
| Caan 2024, Brown 2024 [1, 2] | **L** | Randomization adequate; no material baseline imbalance. | **L** | No important trial-context deviations; analysis matched the assignment effect. | **L** | Outcome data sufficiently complete. | **SC** | Unblinded self-report could be influenced by group awareness; material bias was not established. | **SC** | Outcome not clearly prespecified; result-based selection could not be excluded. | **SC** | SC in D4, D5; no domain was high risk. |
| TrigueroCánovas 2023 [3] | **SC** | Allocation concealment was insufficiently reported. | **L** | No important trial-context deviations; analysis matched the assignment effect. | **SC** | Some data were missing; dependence on true values could not be excluded. | **L** | Measurement appropriate and unlikely to differ between groups. | **SC** | Outcome not clearly prespecified; result-based selection could not be excluded. | **SC** | SC in D1, D3, D5; no domain was high risk. |
| Molenaar 2023 [4] | **L** | Randomization adequate; no material baseline imbalance. | **L** | No important trial-context deviations; analysis matched the assignment effect. | **SC** | Some data were missing; dependence on true values could not be excluded. | **SC** | Unblinded self-report could be influenced by group awareness; material bias was not established. | **L** | Results matched the available prespecified plan. | **SC** | SC in D3, D4; no domain was high risk. |
| Leach 2023 [5] | **L** | Randomization adequate; no material baseline imbalance. | **L** | No important trial-context deviations; analysis matched the assignment effect. | **SC** | Some data were missing; dependence on true values could not be excluded. | **L** | Measurement appropriate and unlikely to differ between groups. | **SC** | No verifiable analysis plan; result-based selection could not be excluded. | **SC** | SC in D3, D5; no domain was high risk. |
| Brown 2023, 2018 CCC, 2018 EC, 2018 P, 2018 PO,2017 [6-11] | **SC** | Allocation concealment was insufficiently reported. | **L** | No important trial-context deviations; analysis matched the assignment effect. | **SC** | Incomplete data; whether missingness depended on true values was unclear. | **SC** | Unblinded self-report could be influenced by group awareness; material bias was not established. | **SC** | Outcome not clearly prespecified; result-based selection could not be excluded. | **SC** | SC in D1, D3, D4, D5; no domain was high risk. |
| Bojesen 2023 [12] | **L** | Randomization adequate; no material baseline imbalance. | **L** | No important trial-context deviations; analysis matched the assignment effect. | **L** | Outcome data sufficiently complete. | **SC** | Unblinded effort-dependent testing could be influenced by guidance; material bias was not established. | **SC** | No verifiable analysis plan; result-based selection could not be excluded. | **SC** | SC in D4, D5; no domain was high risk. |
| Hwang 2022 [13] | **SC** | Random sequence generation and allocation concealment were insufficiently reported. | **H** | Post-intervention response was used to select participants, likely biasing the effect estimate. | **H** | Extensive missing data likely depended on true values and materially biased the results. | **SC** | Unblinded effort-dependent testing could be influenced by guidance; material bias was not established. | **SC** | No verifiable analysis plan; result-based selection could not be excluded. | **H** | High risk in D2, D3; additional concerns in D1, D4, D5. |
| Waller 2020 [14] | **L** | Randomization adequate; no material baseline imbalance. | **L** | No important trial-context deviations; analysis matched the assignment effect. | **L** | Outcome data sufficiently complete. | **SC** | Unblinded self-report and effort-dependent testing could be influenced by group awareness; material bias was not established. | **SC** | No verifiable analysis plan; result-based selection could not be excluded. | **SC** | SC in D4, D5; no domain was high risk. |
| Northgraves 2020 [15] | **L** | Randomization adequate; no material baseline imbalance. | **L** | No important trial-context deviations; analysis matched the assignment effect. | **L** | Outcome data sufficiently complete. | **SC** | Unblinded effort-dependent testing could be influenced by guidance; material bias was not established. | **SC** | No verifiable analysis plan; result-based selection could not be excluded. | **SC** | SC in D4, D5; no domain was high risk. |
| Shim 2019 [16] | **H** | Predictable fixed-order allocation was used. | **L** | No important trial-context deviations; analysis matched the assignment effect. | **H** | Extensive missing data likely depended on true values and materially biased the results. | **L** | Measurement appropriate and unlikely to differ between groups. | **SC** | No verifiable analysis plan; result-based selection could not be excluded. | **H** | High risk in D1, D3; additional concerns in D5. |
| Moug 2019 [17] | **L** | Randomization adequate; no material baseline imbalance. | **L** | No important trial-context deviations; analysis matched the assignment effect. | **SC** | Some data were missing; dependence on true values could not be excluded. | **SC** | Unblinded self-report could be influenced by group awareness; material bias was not established. | **SC** | No verifiable analysis plan; result-based selection could not be excluded. | **SC** | SC in D3, D4, D5; no domain was high risk. |
| Kim 2019 [18] | **SC** | Allocation concealment was insufficiently reported. | **L** | No important trial-context deviations; analysis matched the assignment effect. | **SC** | Some data were missing; dependence on true values could not be excluded. | **SC** | Unblinded self-report could be influenced by group awareness; material bias was not established. | **SC** | No verifiable analysis plan; result-based selection could not be excluded. | **H** | Concerns in D1, D3, D4, D5 accumulated within the same outcome and materially reduced confidence. |
| Gillis 2019 [19] | **L** | Randomization adequate; no material baseline imbalance. | **L** | No important trial-context deviations; analysis matched the assignment effect. | **L** | Outcome data sufficiently complete. | **L** | Measurement appropriate and unlikely to differ between groups. | **H** | Post hoc pooling allowed multiple choices of time points, outcome definitions, and models, making selective reporting likely. | **H** | High risk in D5. |
| Christensen 2019 [20] | **L** | Randomization adequate; no material baseline imbalance. | **L** | No important trial-context deviations; analysis matched the assignment effect. | **SC** | Some data were missing; dependence on true values could not be excluded. | **SC** | Unblinded self-report could be influenced by group awareness; material bias was not established. | **L** | Results matched the available prespecified plan. | **SC** | SC in D3, D4; no domain was high risk. |
| Zimmer 2018 [21] | **SC** | Randomization procedures were insufficiently reported. | **L** | No important trial-context deviations; analysis matched the assignment effect. | **SC** | Some data were missing; dependence on true values could not be excluded. | **SC** | Unblinded self-report and effort-dependent testing could be influenced by group awareness; material bias was not established. | **SC** | No verifiable analysis plan; result-based selection could not be excluded. | **H** | Concerns in D1, D3, D4, D5 accumulated within the same outcome and materially reduced confidence. |
| Lee 2018 [22] | **SC** | Allocation concealment was insufficiently reported. | **L** | No important trial-context deviations; analysis matched the assignment effect. | **SC** | Some data were missing; dependence on true values could not be excluded. | **SC** | Unblinded effort-dependent testing could be influenced by guidance; material bias was not established. | **SC** | No verifiable analysis plan; result-based selection could not be excluded. | **SC** | SC in D1, D3, D4, D5; no domain was high risk. |
| BousquetDion 2018 [23] | **L** | Randomization adequate; no material baseline imbalance. | **L** | No important trial-context deviations; analysis matched the assignment effect. | **H** | Extensive missing data likely depended on true values and materially biased the results. | **L** | Measurement appropriate and unlikely to differ between groups. | **SC** | No verifiable analysis plan; result-based selection could not be excluded. | **H** | High risk in D3; additional concerns in D5. |
| Lee 2017 [24] | **SC** | Allocation concealment was insufficiently reported. | **L** | No important trial-context deviations; analysis matched the assignment effect. | **SC** | Some data were missing; dependence on true values could not be excluded. | **SC** | Unblinded effort-dependent testing could be influenced by guidance; material bias was not established. | **SC** | Outcome not clearly prespecified; result-based selection could not be excluded. | **SC** | SC in D1, D3, D4, D5; no domain was high risk. |
| CantareroVillanueva 2016,2017 [25, 26] | **L** | Randomization adequate; no material baseline imbalance. | **L** | No important trial-context deviations; analysis matched the assignment effect. | **L** | Outcome data sufficiently complete. | **L** | Measurement appropriate and unlikely to differ between groups. | **SC** | No verifiable analysis plan; result-based selection could not be excluded. | **SC** | SC in D5; no domain was high risk. |
| VanVulpen 2016 [27] | **L** | Randomization adequate; no material baseline imbalance. | **L** | No important trial-context deviations; analysis matched the assignment effect. | **L** | Outcome data sufficiently complete. | **SC** | Unblinded self-report could be influenced by group awareness; material bias was not established. | **L** | Results matched the available prespecified plan. | **SC** | SC in D4; no domain was high risk. |
| Courneya 2016 [28] | **SC** | Allocation concealment was insufficiently reported. | **L** | No important trial-context deviations; analysis matched the assignment effect. | **SC** | Some data were missing; dependence on true values could not be excluded. | **SC** | Unblinded effort-dependent testing could be influenced by guidance; material bias was not established. | **L** | Results matched the available prespecified plan. | **SC** | SC in D1, D3, D4; no domain was high risk. |
| Gillis 2014 [29] | **L** | Randomization adequate; no material baseline imbalance. | **L** | No important trial-context deviations; analysis matched the assignment effect. | **L** | Outcome data sufficiently complete. | **SC** | Unblinded self-report could be influenced by group awareness; material bias was not established. | **L** | Results matched the available prespecified plan. | **SC** | SC in D4; no domain was high risk. |
| Pinto 2013 [30] | **SC** | Allocation concealment was insufficiently reported. | **L** | No important trial-context deviations; analysis matched the assignment effect. | **L** | Outcome data sufficiently complete. | **SC** | Unblinded self-report could be influenced by group awareness; material bias was not established. | **SC** | No verifiable analysis plan; result-based selection could not be excluded. | **SC** | SC in D1, D4, D5; no domain was high risk. |
| Bourke 2011 [31] | **L** | Randomization adequate; no material baseline imbalance. | **L** | No important trial-context deviations; analysis matched the assignment effect. | **L** | Outcome data sufficiently complete. | **SC** | Unblinded self-report could be influenced by group awareness; material bias was not established. | **SC** | No verifiable analysis plan; result-based selection could not be excluded. | **SC** | SC in D4, D5; no domain was high risk. |
| Kim 2009 [32] | **SC** | Allocation concealment was insufficiently reported. | **L** | No important trial-context deviations; analysis matched the assignment effect. | **SC** | Some data were missing; dependence on true values could not be excluded. | **SC** | Unblinded effort-dependent testing could be influenced by guidance; material bias was not established. | **SC** | No verifiable analysis plan; result-based selection could not be excluded. | **SC** | SC in D1, D3, D4, D5; no domain was high risk. |
| Courneya 2003 [33] | **SC** | Allocation concealment was insufficiently reported. | **H** | Substantial control-group contamination likely affected the outcomes. | **L** | Outcome data sufficiently complete. | **SC** | Unblinded self-report could be influenced by group awareness; material bias was not established. | **SC** | Outcome not clearly prespecified; result-based selection could not be excluded. | **H** | High risk in D2; additional concerns in D1, D4, D5. |

Note: D1, bias arising from the randomization process; D2, bias due to deviations from intended interventions; D3, bias due to missing outcome data; D4, bias in measurement of the outcome; D5, bias in selection of the reported result. L, low risk of bias; SC, some concerns; H, high risk of bias. For each study, the most severe judgment across the assessed outcomes was reported for each domain and for overall risk of bias.

Fig. S3. Risk of bias assessments by outcome domain

Fig. S4. Funnel plot for assessing publication bias in the overall analysis

Fig. S5. Funnel plots for assessing publication bias by outcome domain

**Supplementary information 7. Extraction Strategy for Moderator Variables.**

Table S4. Moderating variable information summary

| NO. | Name | Extraction Strategy |
| --- | --- | --- |
| (a) Exercise Prescription Moderator Variables | | |
| 1 | Type (AE/RT/PA) | AE is aerobic exercise. RT is resistance exercise. PA is a recommendation for increasing physical activity. |
| 2 | Duration | Duration is the duration of the exercise intervention. Only pre- and post-intervention values are included. |
| 3 | Weekly Times | Weekly Times refers to the number of exercise interventions per week. |
| 4 | Single Exercise Intensity | HR_max_/HRR (Heart Rate Reserve): 50%–70% = 3–6 MET, 70%–85% = 6–10 MET, >85% = 10 MET.  If the intensity of RT is not clearly marked, it is calculated according to 4 MET.  Borg scale: 12–20 = 4–10 MET.  Single Exercise Intensity = MET × Single exercise duration (excludes warm-up and stretching/cool-down). |
| 5 | Weekly Exercise Volume | Weekly Exercise Volume = Single Exercise Intensity × Weekly Times |
| 6 | Supervision (Yes/Half/No) | Half is combination of supervised and unsupervised intervention. |
| 7 | Other Intervention (Yes/No) |  |
| (b) Background Moderator Variables | | |
| 1 | Timing (Preoperative/Survivor/Chemotherapy) | Preoperative is a study of preoperative rehabilitation. Survivor is a study of colorectal cancer survivors. Chemotherapy is a study conducted during chemotherapy. |
| 2 | Age | Age is the mean age of the intervention group and the control group. |
| 3 | Male Ratio | Male Ratio is the proportion of male patients in the study. |
| 4 | BMI | BMI is the mean body mass index of the intervention group and the control group. |
| 5 | Weight | Weight is the mean weight of the intervention group and the control group. |
| 6 | Colon Cancer Ratio | Colon Cancer Ratio is the proportion of patients with colon cancer in the study. |
| 7 | Cancer Stage (0-II) | Cancer Stage (0-II) is the proportion of 0-II cancer patients in the study determined by clinical examinations (such as imaging examinations, physical examinations, etc.) and pathological examinations (such as histology, cytology, etc.). |
| 8 | Continents (Europe/North America/Asia) | Continents are divided according to the continent where the experimental country is located |
| 9 | Race* |  |
| 10 | Surgery Type* |  |
| 11 | Chemotherapy Regimen* |  |
| 12 | Comorbidity (Diabetes)* |  |
| 13 | Comorbidity (Cardiovascular disease)* |  |
| 14 | Comorbidity (Pulmonary disease)* |  |
| 15 | Smoking Status* |  |
| 16 | Drinking Status* |  |
| 17 | Other symptoms (Sarcopenia)* |  |
| 18 | Other symptoms (Weakness)* |  |

Note: The asterisk (*) denotes the excluded moderator variables.

Table S5. Frequency statistics of the moderating variables

| No. | Variables Name | Median/N | Interquartile range (Q1–Q3) |
| --- | --- | --- | --- |
| (a) Exercise Prescription Moderator Variables | | | |
| 1 | Type (AE/RT/PA/AE+RT) |  |  |
|  | AE | 87 |  |
|  | RT | 29 |  |
|  | PA | 18 |  |
|  | AE+RT | 132 |  |
| 2 | Duration | 12 | 6-24 |
| 3 | Weekly Times | 4 | 3-7 |
| 4 | Single Exercise Intensity | 158 | 150-216 |
| 5 | Weekly Exercise Volume | 635 | 592-1056 |
| 6 | Supervision (Yes/Half/No) |  |  |
|  | Yes | 68 |  |
|  | Half | 86 |  |
|  | No | 112 |  |
| 7 | Other Intervention (Yes/No) |  |  |
|  | Yes | 61 |  |
|  | No | 205 |  |
| (b) Background Moderator Variables | | | |
| 1 | Timing (Preoperative/Chemotherapy/Survivor) |  |  |
|  | Preoperative | 48 |  |
|  | Chemotherapy | 58 |  |
|  | Survivor | 160 |  |
| 2 | Age | 58 | 56-60 |
| 3 | Male Ratio | 0.5 | 0.44-0.58 |
| 4 | BMI | 27.5 | 26.3-28.99 |
| 5 | Weight | 76.2 | 62.98-80.72 |
| 6 | Colon Cancer Ratio | 0.82 | 0.64-1 |
| 7 | Cancer Stage (0-II) | 0.49 | 0.13-0.53 |
| 8 | Continents (Europe/North America/Asia) |  |  |
|  | Europe | 99 |  |
|  | North America | 121 |  |
|  | Asia | 46 |  |

Note: AE is aerobic exercise. RT is resistance exercise. PA is a recommendation for increasing physical activity.

**Supplementary information 8. Results of the RVE Univariate Regression Model.**

| Model | labels | b.r | SE | t | dfs | prob | CI.L | CI.U | sig | I^2^ (%) | R^2^ |
| --- | --- | --- | --- | --- | --- | --- | --- | --- | --- | --- | --- |
| (a) Exercise Prescription Moderator Variables | | | | | | | | | | | |
| CRF | X.Intercept. | 0.537 | 0.049 | 11.026 | 1.313 | 0.030 | 0.178 | 0.896 | ** | 4.87% | 0.16 |
|  | Supervision.No | -0.195 | 0.117 | -1.668 | 3.241 | 0.187 | -0.551 | 0.162 |  |  |  |
|  | Supervision.Yes | -0.283 | 0.080 | -3.521 | 2.659 | 0.047 | -0.559 | -0.008 | ** |  |  |
| MF | X.Intercept. | -0.088 | 0.225 | -0.389 | 8.840 | 0.707 | -0.598 | 0.423 |  | 45.4% | 0.07 |
|  | Weekly.Times | 0.119 | 0.059 | 2.027 | 8.771 | 0.074 | -0.014 | 0.253 | * |  |  |
| (b) Background Moderator Variables | | | | | | | | | | | |
| BC | X.Intercept. | -0.201 | 0.070 | -2.883 | 4.169 | 0.043 | -0.392 | -0.010 | ** | 0 | 0 |
|  | Male.Ratio | 0.485 | 0.123 | 3.929 | 3.750 | 0.019 | 0.133 | 0.836 | ** |  |  |
| CRF | X.Intercept. | 0.075 | 0.000 | 5039243.502 | 4.200 | 0.000 | 0.075 | 0.075 | *** | 3.47% | 0.41 |
|  | Timing.Preoperative | 0.185 | 0.060 | 3.072 | 1.618 | 0.118 | -0.143 | 0.514 |  |  |  |
|  | Timing.Survivor | 0.367 | 0.072 | 5.090 | 4.047 | 0.007 | 0.168 | 0.567 | *** |  |  |
| MF | X.Intercept. | 2.371 | 0.813 | 2.916 | 9.452 | 0.016 | 0.545 | 4.197 | ** | 40.87% | 0.23 |
|  | Weight | -0.027 | 0.011 | -2.475 | 10.031 | 0.033 | -0.052 | -0.003 | ** |  |  |
| MF | X.Intercept. | 1.023 | 0.192 | 5.341 | 2.933 | 0.014 | 0.405 | 1.640 | ** | 41.16% | 0.22 |
|  | Colon.Cancer.Ratio | -0.811 | 0.231 | -3.514 | 3.226 | 0.035 | -1.517 | -0.105 | ** |  |  |

**Supplementary information 9. Results of the MetaForest Regression Model.**

Table S6. Parameters of the MetaForest Regression Model

| ID | R²_OOB_ | τ² | Feature Weights | Mtry | min.node.size | R²_CV_ | R²_CV_ SD | RMSE | RMSE SD | MAE | MAE SD |
| --- | --- | --- | --- | --- | --- | --- | --- | --- | --- | --- | --- |
| (a) Exercise Prescription Moderator Variables | | | | | | | | | | | |
| BC | -0.13 | 0.00 | random | 2 | 4 | 0.04 | 0.06 | 0.22 | 0.06 | 0.14 | 0.03 |
| CAB | -0.07 | 0.00 | fixed | 2 | 4 | 0.06 | 0.08 | 0.42 | 0.08 | 0.31 | 0.06 |
| CRF | 0.04 | 0.05 | unif | 2 | 4 | 0.24 | 0.20 | 0.27 | 0.05 | 0.23 | 0.05 |
| MF | -0.05 | 0.11 | fixed | 2 | 4 | 0.08 | 0.09 | 0.56 | 0.14 | 0.43 | 0.09 |
| PFC | 0.02 | 0.00 | fixed | 2 | 4 | 0.18 | 0.23 | 0.71 | 0.54 | 0.40 | 0.25 |
| HRQoL | -0.11 | 0.05 | fixed | 2 | 2 | 0.04 | 0.07 | 0.37 | 0.08 | 0.26 | 0.04 |
| (b) Background Moderator Variables | | | | | | | | | | | |
| BC | -0.13 | 0.00 | random | 2 | 4 | 0.05 | 0.07 | 0.22 | 0.06 | 0.14 | 0.03 |
| CAB | -0.03 | 0.00 | fixed | 2 | 4 | 0.07 | 0.07 | 0.41 | 0.08 | 0.30 | 0.06 |
| CRF | 0.08 | 0.07 | unif | 2 | 4 | 0.25 | 0.21 | 0.26 | 0.05 | 0.22 | 0.05 |
| MF | -0.07 | 0.11 | fixed | 2 | 4 | 0.09 | 0.10 | 0.56 | 0.13 | 0.42 | 0.09 |
| PFC | 0.03 | 0.00 | fixed | 2 | 4 | 0.18 | 0.24 | 0.72 | 0.53 | 0.40 | 0.25 |
| HRQoL | -0.15 | 0.05 | fixed | 2 | 4 | 0.04 | 0.05 | 0.37 | 0.08 | 0.27 | 0.04 |

Note: R^2^_OOB_: Out-of-Bag R-Squared. τ²: Between-Studies Variance. R^2^_CV_: Cross-Validation R-Squared. R^2^_CV_ SD: Cross-Validation R-Squared Standard Deviation. Mtry: Number of Variables Randomly Sampled as Candidates at Each Split. min.node.size: Minimum Node Size. RMSE: Root Mean Square Error. MASE: Mean Absolute Scaled Error.

Fig. S6. Convergence plot of the metaforest model with exercise prescription moderator variables

Fig. S7. Convergence plot of the metaforest model with background moderator variables

Fig. S8. Partial dependence plot (exercise prescription moderator variables of BC)

Fig. S9. Partial dependence plot (exercise prescription moderator variables of CAB)

Fig. S10. Partial dependence plot (exercise prescription moderator variables of CRF)

Fig. S11. Partial dependence plot (exercise prescription moderator variables of MF)

Fig. S12. Partial dependence plot (exercise prescription moderator variables of PFC)

Fig. S13. Partial dependence plot (exercise prescription moderator variables of HRQoL)

Fig. S14. Heatmap summarizing variable importance generated by MetaForest model of background moderator variables

Fig. S15. Partial dependence plot (background moderator variables of BC)

Fig. S16. Partial dependence plot (background moderator variables of CAB)

Fig. S17. Partial dependence plot (background moderator variables of CRF)

Fig. S18. Partial dependence plot (background moderator variables of MF)

Fig. S19. Partial dependence plot (background moderator variables of PFC)

Fig. S20. Partial dependence plot (background moderator variables of HRQoL)

**Supplementary information 10. Results of Subgroup analysis.**

Fig. S21. Forest plot of summary effect of subgroup analysis by timing

Table S7. RVE model parameters without covariates for subgroup analysis

|  | ES | SE | t | dfs | prob | CI.L | CI.U | sig | I² | V mean |
| --- | --- | --- | --- | --- | --- | --- | --- | --- | --- | --- |
| Chemotherapy | 0.148 | 0.105 | 1.407 | 3.025 | 0.254 | -0.185 | 0.482 |  | 22.91% | 0.097 |
| Preoperative | 0.242 | 0.065 | 3.729 | 6.355 | 0.009 | 0.085 | 0.398 | *** | 30.54% | 0.107 |
| Survivor | 0.179 | 0.034 | 5.342 | 10.251 | 0.000 | 0.105 | 0.254 | *** | 38.89% | 0.159 |

Table S8. Parameters of the MetaForest regression model for subgroup analysis

| ID | R²_OOB_ | τ² | Feature Weights | Mtry | min.node.size | R²_CV_ | R²_CV_ SD | RMSE | RMSE SD | MAE | MAE SD |
| --- | --- | --- | --- | --- | --- | --- | --- | --- | --- | --- | --- |
| (a) Exercise Prescription Moderator Variables | | | | | | | | | | | |
| Chemotherapy | 0.06 | 0.00 | fixed | 2 | 4 | 0.10 | 0.09 | 0.32 | 0.06 | 0.24 | 0.04 |
| Preoperative | -0.06 | 0.02 | fixed | 2 | 4 | 0.08 | 0.10 | 0.41 | 0.15 | 0.26 | 0.07 |
| Survivor | -0.07 | 0.05 | fixed | 2 | 4 | 0.03 | 0.04 | 0.52 | 0.12 | 0.33 | 0.04 |
| (b) Background Moderator Variables | | | | | | | | | | | |
| Chemotherapy | 0.06 | 0.00 | fixed | 2 | 4 | 0.11 | 0.09 | 0.32 | 0.06 | 0.24 | 0.04 |
| Preoperative | -0.05 | 0.02 | fixed | 2 | 4 | 0.07 | 0.10 | 0.42 | 0.15 | 0.27 | 0.07 |
| Survivor | -0.07 | 0.06 | fixed | 2 | 4 | 0.03 | 0.04 | 0.52 | 0.12 | 0.33 | 0.04 |

Table S9. Overview of variable importance generated by metaforest models in subgroup analysis

| ID | Chemotherapy | | Preoperative | | Survivor | |
| --- | --- | --- | --- | --- | --- | --- |
|  | Importance | p | Importance | p | Importance | p |
| (a) Exercise Prescription Moderator Variables | | | | | | |
| Type | 0.0031 | 0.0891 | 0.0040 | 0.0990 | -0.0102 | 0.9901 |
| Duration | 0.0071 | **0.0198** | 0.0000 | 1.0000 | -0.0072 | 0.9703 |
| Weekly Times | 0.0015 | 0.1386 | 0.0020 | 0.4950 | -0.0002 | 0.7822 |
| Single Exercise Intensity | 0.0047 | 0.0891 | -0.0067 | 0.9307 | -0.0052 | 0.9208 |
| Weekly Exercise Volume | 0.0055 | **0.0495** | 0.0046 | 0.3762 | -0.0055 | 0.9703 |
| Supervision | 0.0018 | 0.1485 | -0.0008 | 0.6931 | -0.0029 | 0.9307 |
| Other Intervention | 0.0010 | 0.1782 | 0.0016 | 0.3069 | -0.0026 | 0.9604 |
| (b) Background Moderator Variables | | | | | | |
| Age | 0.0042 | 0.0693 | 0.0017 | 0.4455 | -0.0020 | 0.8812 |
| Male Ratio | 0.0031 | **0.0495** | 0.0045 | 0.1188 | -0.0016 | 0.9208 |
| BMI | 0.0066 | **0.0099** | -0.0005 | 0.4653 | -0.0030 | 0.9703 |
| Weight | 0.0020 | 0.1188 | 0.0038 | 0.1980 | -0.0033 | 0.9802 |
| Colon Cancer Ratio | 0.0037 | **0.0495** | 0.0072 | 0.1287 | -0.0062 | 0.9901 |
| Cancer Stage | 0.0039 | **0.0495** | 0.0013 | 0.3564 | -0.0081 | 1.0000 |
| Continents | 0.0014 | 0.1287 | 0.0029 | 0.2178 | -0.0017 | 0.9604 |

Note: Bold fonts indicate p < 0.05.

Fig. S22. Partial dependence plot (chemotherapy: exercise prescription moderator variables of overall effect size)

Fig. S23. Partial dependence plot (preoperative: exercise prescription moderator variables of overall effect size)

Fig. S24. Partial dependence plot (survivor: exercise prescription moderator variables of overall effect size)

Fig. S25. Partial dependence plot (chemotherapy: background moderator variables of overall effect size)

Fig. S26. Partial dependence plot (preoperative: background moderator variables of overall effect size)

Fig. S27. Partial dependence plot (survivor: background moderator variables of overall effect size)

**Supplementary information 11. Grading of Recommendations, Assessment, Development and Evaluations (GRADE).**

Table S10. GRADE rating for effect size

| Outcome | Effect Size | SE | Risk of Bias | Imprecision | Indirectness | Inconsistency (I²) | Publication Bias | GRADE Quality |
| --- | --- | --- | --- | --- | --- | --- | --- | --- |
| Body Composition (BC) | 0.06 | 0.021 | Low n=2  Some concerns n=7  High n=4** | 0.035 | Direct | 0 | Egger’s p = 0.19 | Low** |
| Cancer-Associated Biomarkers (CAB) | 0.16 | 0.094 | Some concerns n=4  High n=2** | 0.178† | Direct | 0 | Egger’s p = 0.22 | Very Low**† |
| Cardiorespiratory Fitness (CRF) | 0.35 | 0.058 | Low n=1  Some concerns n=10  High n=1* | 0.001 | Direct | 5.74% | Egger’s p = 0.27 | Moderate* |
| Muscula Fitness (MF) | 0.45 | 0.104 | Low n=2  Some concerns n=14  High n=2* | 0.001 | Direct | 47.20%† | Egger’s p = 0.14 | Low*† |
| Physical Function Capacity (PFC) | 0.22 | 0.148 | Some concerns n=10  High n=1* | 0.179† | Direct | 55.19%† | Egger’s p = 0.09 | Very Low*‡ |
| Health-Related Quality of Life (HRQoL) | 0.03 | 0.062 | Some concerns n=11  High n=3* | 0.611† | Direct | 32.09%† | Egger’s p = 0.77 | Very Low*‡ |

Note: Reasons for downgrading: †Imprecision (1 downgrade), ‡Severe imprecision (2 downgrades), *risk of bias (1 downgrade) , #inconsistency (1 downgrade).

Table S11. GRADE rating for Metaforest

| Outcome | Risk of Bias | Imprecision(p) | Inconsistency | Indirectness | Publication Bias | GRADE Quality |
| --- | --- | --- | --- | --- | --- | --- |
| BC | Low n=2  Some concerns n=7  High n=4** | No | Direct | † | Egger’s p = 0.19 | Very Low**† |
| CAB | Some concerns n=4  High n=2** | No | Direct | † | Egger’s p = 0.22 | Very Low**† |
| CRF | Low n=1  Some concerns n=10  High n=1* | No | Direct |  | Egger’s p = 0.27 | Low* |
| MF | Low n=2  Some concerns n=14  High n=2* | No | Direct |  | Egger’s p = 0.14 | Low* |
| PFC | Some concerns n=10  High n=1* | No | Direct | † | Egger’s p = 0.09 | Very Low*† |
| HRQoL | Some concerns n=11  High n=3* | No | Direct | † | Egger’s p = 0.77 | Very Low*† |

Note: The initial rating was assigned as “Moderate.” Inconsistency was assessed based on model performance differences across the three categories of moderator variables (RMSE, MAE, RMSE SD, and MAE SD). Imprecision was evaluated based on variation in model performance across the three moderator categories (R²CV and R²CV SD). †Imprecision (1 downgrade), ‡Severe imprecision (2 downgrades), *risk of bias (1 downgrade) , #inconsistency (1 downgrade).

**References**

1.	Caan BJ, Brown JC, Lee C, Binder AM, Weltzien E, Ross MC, et al. Effect of home-based resistance training on chemotherapy relative dose intensity and tolerability in colon cancer: The FORCE randomized control trial. Cancer Name. 2024; <https://doi.org/10.1002/cncr.35204>

2.	Brown JC, Yang S, Compton SLE, Campbell KL, Cespedes Feliciano EM, Quinney S, et al. Effect of resistance training on physical function during chemotherapy in colon cancer. JNCI Cancer Spectr Name. 2024; <https://doi.org/10.1093/jncics/pkae058>

3.	Triguero-Cánovas D, López-Rodríguez-Arias F, Gómez-Martínez M, Sánchez-Guillén L, Peris-Castelló F, Alcaide-Quirós MJ, et al. Home-based prehabilitation improves physical conditions measured by ergospirometry and 6MWT in colorectal cancer patients: a randomized controlled pilot study. Support Care Cancer Name. 2023; <https://doi.org/10.1007/s00520-023-08140-4>

4.	Molenaar CJL, Minnella EM, Coca-Martinez M, Ten Cate DWG, Regis M, Awasthi R, et al. Effect of Multimodal Prehabilitation on Reducing Postoperative Complications and Enhancing Functional Capacity Following Colorectal Cancer Surgery: The PREHAB Randomized Clinical Trial. JAMA Surg Name. 2023; <https://doi.org/10.1001/jamasurg.2023.0198>

5.	Leach HJ, Gomes EL, Hidde MC, Lyden K, Bryan AD, Cockburn MG, et al. A videoconference physical activity intervention for colorectal cancer survivors: a pilot randomized controlled trial. OBM Integrative and Complementary Medicine Name. 2023; <https://doi.org/10.21926/obm.icm.2304060>

6.	Brown JC, Compton SLE, Meyerhardt JA, Spielmann G, Yang S. The dose-response effect of aerobic exercise on inflammation in colon cancer survivors. Front Oncol Name. 2023; <https://doi.org/10.3389/fonc.2023.1257767>

7.	Brown JC, Rickels MR, Troxel AB, Zemel BS, Damjanov N, Ky B, et al. Dose-response effects of exercise on insulin among colon cancer survivors. Endocr Relat Cancer Name. 2018; <https://doi.org/10.1530/erc-17-0377>

8.	Brown JC, Damjanov N, Courneya KS, Troxel AB, Zemel BS, Rickels MR, et al. A randomized dose-response trial of aerobic exercise and health-related quality of life in colon cancer survivors. Psychooncology Name. 2018; <https://doi.org/10.1002/pon.4655>

9.	Brown JC, Rhim AD, Manning SL, Brennan L, Mansour AI, Rustgi AK, et al. Effects of exercise on circulating tumor cells among patients with resected stage I-III colon cancer. PLoS One Name. 2018; <https://doi.org/10.1371/journal.pone.0204875>

10.	Brown JC, Troxel AB, Ky B, Damjanov N, Zemel BS, Rickels MR, et al. Dose-response Effects of Aerobic Exercise Among Colon Cancer Survivors: A Randomized Phase II Trial. Clin Colorectal Cancer Name. 2018; <https://doi.org/10.1016/j.clcc.2017.06.001>

11.	Brown JC, Zemel BS, Troxel AB, Rickels MR, Damjanov N, Ky B, et al. Dose-response effects of aerobic exercise on body composition among colon cancer survivors: a randomised controlled trial. Br J Cancer Name. 2017; <https://doi.org/10.1038/bjc.2017.339>

12.	Bojesen RD, Dalton SO, Skou ST, Jørgensen LB, Walker LR, Eriksen JR, et al. Preoperative multimodal prehabilitation before elective colorectal cancer surgery in patients with WHO performance status I or II: randomized clinical trial. BJS Open Name. 2023; <https://doi.org/10.1093/bjsopen/zrad134>

13.	Hwang SH, Kang DW, Lee MK, Byeon JY, Park H, Park DH, et al. Changes in DNA methylation after 6-week exercise training in colorectal cancer survivors: A preliminary study. Asia Pac J Clin Oncol Name. 2022; <https://doi.org/10.1111/ajco.13482>

14.	E. Waller SR, P. Sutton, J. Allen, J. Saxton and O. Aziz. **Randomised controlled trial of patients undergoing prehabilitation with wearables versus standard of care before major abdominal cancer surgery (Trial Registration: NCT04047524)**. Colorectal Disease Name. 2020; <https://doi.org/10.1111/codi.15168>

15.	Northgraves MJ, Arunachalam L, Madden LA, Marshall P, Hartley JE, MacFie J, et al. Feasibility of a novel exercise prehabilitation programme in patients scheduled for elective colorectal surgery: a feasibility randomised controlled trial. Support Care Cancer Name. 2020; <https://doi.org/10.1007/s00520-019-05098-0>

16.	Shim YJ, Kim HJ, Oh SC, Lee SI, Choi SW. Exercise during adjuvant treatment for colorectal cancer: treatment completion, treatment-related toxicities, body composition, and serum level of adipokines. Cancer Manag Res Name. 2019; <https://doi.org/10.2147/cmar.S208754>

17.	Moug SJ, Mutrie N, Barry SJE, Mackay G, Steele RJC, Boachie C, et al. Prehabilitation is feasible in patients with rectal cancer undergoing neoadjuvant chemoradiotherapy and may minimize physical deterioration: results from the REx trial. Colorectal Dis Name. 2019; <https://doi.org/10.1111/codi.14560>

18.	Kim JY, Lee MK, Lee DH, Kang DW, Min JH, Lee JW, et al. Effects of a 12-week home-based exercise program on quality of life, psychological health, and the level of physical activity in colorectal cancer survivors: a randomized controlled trial. Support Care Cancer Name. 2019; <https://doi.org/10.1007/s00520-018-4588-0>

19.	Gillis C, Fenton TR, Sajobi TT, Minnella EM, Awasthi R, Loiselle S, et al. Trimodal prehabilitation for colorectal surgery attenuates post-surgical losses in lean body mass: A pooled analysis of randomized controlled trials. Clin Nutr Name. 2019; <https://doi.org/10.1016/j.clnu.2018.06.982>

20.	Christensen JF, Sundberg A, Osterkamp J, Thorsen-Streit S, Nielsen AB, Olsen CK, et al. Interval Walking Improves Glycemic Control and Body Composition After Cancer Treatment: A Randomized Controlled Trial. J Clin Endocrinol Metab Name. 2019; <https://doi.org/10.1210/jc.2019-00590>

21.	Zimmer P, Trebing S, Timmers-Trebing U, Schenk A, Paust R, Bloch W, et al. Eight-week, multimodal exercise counteracts a progress of chemotherapy-induced peripheral neuropathy and improves balance and strength in metastasized colorectal cancer patients: a randomized controlled trial. Support Care Cancer Name. 2018; <https://doi.org/10.1007/s00520-017-3875-5>

22.	Lee MK, Kim NK, Jeon JY. Effect of the 6-week home-based exercise program on physical activity level and physical fitness in colorectal cancer survivors: A randomized controlled pilot study. PLoS One Name. 2018; <https://doi.org/10.1371/journal.pone.0196220>

23.	Bousquet-Dion G, Awasthi R, Loiselle S, Minnella EM, Agnihotram RV, Bergdahl A, et al. Evaluation of supervised multimodal prehabilitation programme in cancer patients undergoing colorectal resection: a randomized control trial. Acta Oncol Name. 2018; <https://doi.org/10.1080/0284186x.2017.1423180>

24.	Lee MK, Kim JY, Kim DI, Kang DW, Park JH, Ahn KY, et al. Effect of home-based exercise intervention on fasting insulin and Adipocytokines in colorectal cancer survivors: a randomized controlled trial. Metabolism Name. 2017; <https://doi.org/10.1016/j.metabol.2017.07.005>

25.	Cantarero-Villanueva I, Sánchez-Jiménez A, Galiano-Castillo N, Díaz-Rodríguez L, Martín-Martín L, Arroyo-Morales M. Effectiveness of Lumbopelvic Exercise in Colon Cancer Survivors: A Randomized Controlled Clinical Trial. Med Sci Sports Exerc Name. 2016; <https://doi.org/10.1249/mss.0000000000000917>

26.	Cantarero-Villanueva I, Cuesta-Vargas AI, Lozano-Lozano M, Fernández-Lao C, Fernández-Pérez A, Galiano-Castillo N. Changes in Pain and Muscle Architecture in Colon Cancer Survivors After a Lumbopelvic Exercise Program: A Secondary Analysis of a Randomized Controlled Trial. Pain Med Name. 2017; <https://doi.org/10.1093/pm/pnx026>

27.	Van Vulpen JK, Velthuis MJ, Steins Bisschop CN, Travier N, Van Den Buijs BJ, Backx FJ, et al. Effects of an Exercise Program in Colon Cancer Patients undergoing Chemotherapy. Med Sci Sports Exerc Name. 2016; <https://doi.org/10.1249/mss.0000000000000855>

28.	Courneya KS, Vardy JL, O'Callaghan CJ, Friedenreich CM, Campbell KL, Prapavessis H, et al. Effects of a Structured Exercise Program on Physical Activity and Fitness in Colon Cancer Survivors: One Year Feasibility Results from the CHALLENGE Trial. Cancer Epidemiol Biomarkers Prev Name. 2016; <https://doi.org/10.1158/1055-9965.Epi-15-1267>

29.	Gillis C, Li C, Lee L, Awasthi R, Augustin B, Gamsa A, et al. Prehabilitation versus rehabilitation: a randomized control trial in patients undergoing colorectal resection for cancer. Anesthesiology Name. 2014; <https://doi.org/10.1097/aln.0000000000000393>

30.	Pinto BM, Papandonatos GD, Goldstein MG, Marcus BH, Farrell N. Home-based physical activity intervention for colorectal cancer survivors. Psychooncology Name. 2013; <https://doi.org/10.1002/pon.2047>

31.	Bourke L, Thompson G, Gibson DJ, Daley A, Crank H, Adam I, et al. Pragmatic lifestyle intervention in patients recovering from colon cancer: a randomized controlled pilot study. Arch Phys Med Rehabil Name. 2011; <https://doi.org/10.1016/j.apmr.2010.12.020>

32.	Kim DJ, Mayo NE, Carli F, Montgomery DL, Zavorsky GS. Responsive measures to prehabilitation in patients undergoing bowel resection surgery. Tohoku J Exp Med Name. 2009; <https://doi.org/10.1620/tjem.217.109>

33.	Courneya KS, Friedenreich CM, Quinney HA, Fields AL, Jones LW, Fairey AS. A randomized trial of exercise and quality of life in colorectal cancer survivors. Eur J Cancer Care (Engl) Name. 2003; <https://doi.org/10.1046/j.1365-2354.2003.00437.x>
